# Emodepside targets SLO-1 channels of *Onchocerca ochengi* and induces broad anthelmintic effects in a bovine model of onchocerciasis

**DOI:** 10.1371/journal.ppat.1009601

**Published:** 2021-06-02

**Authors:** Germanus S. Bah, Sebastian Schneckener, Steffen R. Hahnel, Nicolas H. Bayang, Helena Fieseler, Gabriele M. Schmuck, Ralph Krebber, Anouk Sarr, Carsten Terjung, Henrietta F. Ngangyung, David D. Ekale, Youssouf M. Mfopit, Lucien Rufener, John Graham-Brown, Vincent N. Tanya, Martin Glenschek-Sieberth, Daniel Kulke, Benjamin L. Makepeace

**Affiliations:** 1 Institut de Recherche Agricole pour le Développement, Centre Régional de Wakwa, Ngaoundéré, Cameroun; 2 Bayer AG, Engineering & Technology, Applied Mathematics, Leverkusen, Germany; 3 Bayer Animal Health GmbH, Drug Discovery & External Innovation, Monheim am Rhein, Germany; 4 Bayer AG, Crop Science Division, Research & Development-Regulatory Science-Human Safety-Residue Analysis, Monheim am Rhein, Germany; 5 INVENesis Sarl, St-Blaise (NE), Switzerland; 6 Bayer AG, Pharmaceuticals, DMPK, Wuppertal, Germany; 7 Institute of Infection, Veterinary and Ecological Sciences, University of Liverpool, Liverpool, United Kingdom; 8 Cameroon Academy of Sciences, Yaoundé, Cameroon; 9 Bayer AG, Pharmaceuticals, Wuppertal, Germany; Washington University School of Medicine, UNITED STATES

## Abstract

Onchocerciasis (river blindness), caused by the filarial worm *Onchocerca volvulus*, is a neglected tropical disease mostly affecting sub-Saharan Africa and is responsible for >1.3 million years lived with disability. Current control relies almost entirely on ivermectin, which suppresses symptoms caused by the first-stage larvae (microfilariae) but does not kill the long-lived adults. Here, we evaluated emodepside, a semi-synthetic cyclooctadepsipeptide registered for deworming applications in companion animals, for activity against adult filariae (*i*.*e*., as a macrofilaricide). We demonstrate the equivalence of emodepside activity on SLO-1 potassium channels in *Onchocerca volvulus* and *Onchocerca ochengi*, its sister species from cattle. Evaluation of emodepside in cattle as single or 7-day treatments at two doses (0.15 and 0.75 mg/kg) revealed rapid activity against microfilariae, prolonged suppression of female worm fecundity, and macrofilaricidal effects by 18 months post treatment. The drug was well tolerated, causing only transiently increased blood glucose. Female adult worms were mostly paralyzed; however, some retained metabolic activity even in the multiple high-dose group. These data support ongoing clinical development of emodepside to treat river blindness.

## Author summary

Onchocerciasis (river blindness), caused by the parasitic worm *Onchocerca volvulus*, is a devastating neglected tropical disease affecting sub-Saharan Africa with an overall impact of >1.3 million years lived with disability. Current control relies mainly on a single drug, ivermectin, which suppresses symptoms caused by the first-stage larvae (microfilariae) but does not kill the long-lived adults. The identification of a drug that can safely eliminate adult worms (*i*.*e*., a macrofilaricide) is a major research objective for onchocerciasis. We evaluated the anthelminthic activity of emodepside, a veterinary wormer, in cattle infected with a close relative of *O*. *volvulus* (*Onchocerca ochengi*) before conducting pharmacokinetic modelling to estimate drug distribution in humans. Emodepside as single or 7-day treatments at two doses produced rapid activity against *O*. *ochengi* microfilariae, prolonged suppression of female worm fecundity, and paralysed most female worms by 18 months, although some remained metabolically active even in the multiple high-dose treatment group. The drug was well tolerated, causing only transiently increased blood glucose. Thus, emodepside shows slow but significant efficacy against adult *O*. *ochengi* in naturally infected cattle, meeting the criteria for a safe macrofilaricidal drug. Our data support the ongoing clinical development of emodepside for the treatment of human onchocerciasis.

## Introduction

Onchocerciasis is a neglected tropical disease caused by a parasitic worm, *Onchocerca volvulus*, which belongs to the vector-transmitted superfamily Filarioidea (the filarial nematodes). The vector that carries the parasite (blackfly *Simulium* spp.) requires fast-flowing water during its larval stage, and infection with the parasite affects the skin and eyes, causing severe dermatitis and keratitis; hence the disease is colloquially known as river blindness. Onchocerciasis has been the subject of one of the longest-running series of control programmes in the history of tropical medicine, spanning 45 years [1], yet 21 million people remain infected and the current disease burden has been estimated as >1.3 million years lived with disability [2]. The disease is heavily concentrated in sub-Saharan Africa, though hotspots remain in Yemen and Amazonia.

Onchocerciasis control originally focused on the vector, but since the late 1980s this approach has been largely superseded by mass drug administration (MDA) with a single compound, ivermectin (IVM; trade name “Mectizan”) which targets the worm [3]. In most endemic foci in sub-Saharan Africa, IVM is distributed once annually, killing the first-stage larvae (microfilariae, Mf) in the skin and thus preventing disease manifestations. This regimen also suppresses the release of new Mf from the adult female worm for several months [4]. In foci with seasonal transmission, MDA with IVM (which may include semi-annual treatments) has also been demonstrated to be capable of breaking transmission, typically after an interval of 15–25 years [5,6]. The long period of MDA required to abrogate transmission is due to the longevity of the adult worms [7], as the female can be reproductively active for more than 10 years and is only temporarily sterilised, not destroyed, by IVM treatments. Although the current World Health Organization (WHO) strategy is to eliminate onchocerciasis by MDA, modelling suggests that IVM alone cannot achieve elimination until the 2040s rather than 2025 [8], the target date of the Mectizan Donation Program [9]. This sole reliance on IVM has other drawbacks, including the emergence of suboptimal responses to the drug in some *O*. *volvulus* populations in Ghana and Cameroon [10,11] and IVM contraindications in people heavily co-infected with another filarial nematode, *Loa loa*, who are at risk of severe adverse events [12,13].

One of the key goals of onchocerciasis research over several decades has been to identify a drug with a good safety profile that can kill the adult stage of the parasite (a ‘macrofilaricide’). At one time, moxidectin, a drug in the same class as IVM (the macrocyclic lactones), was considered to have macrofilaricidal potential [14]. However, several animal studies, including in the natural *Onchocerca ochengi* system in cattle [15], have failed to demonstrate any significant macrofilaricidal effects of moxidectin, although it suppresses Mf for longer than IVM after a single dose [16,17]. Following successful Phase III trials that supported this superior microfilaricidal and sterilising effect [18], moxidectin has recently been approved by the US Food and Drug Administration for use against onchocerciasis. Recent preclinical studies have provided strong evidence that oxfendazole (a benzimidazole [19]) and ABBV-4083 (a derivative of tylosin—a macrolide antibiotic targeting the obligate *Wolbachia* endosymbiont found in many filarial species [20]) have genuine macrofilaricidal activity. A third leading macrofilaricidal compound, emodepside (a cyclooctadepsipeptide), is the focus of the current study.

The anthelmintic activity of a new *N*-methylated cyclooctadepsipeptide, PF1022A, the parent compound of emodepside, was originally described against *Ascaridia galli* in chickens [21]. PF1022A is a fermentation product of the fungus *Rosellinia* spp., found on the leaves of *Camellia japonica* [22]. While PF1022A treatments of multiple host species resulted in good efficacy against intestinal stages of a variety of nematodes, parenteral stages were not sufficiently affected [23–25]. Therefore, to increase systemic exposure, two morpholine rings were added to the depsipeptide structure, resulting in the semi-synthetic derivative, emodepside. Evaluation of emodepside revealed a broader spectrum of activity *in vivo*, including efficacy against cerebral stages of *Angiostrongylus cantonensis* and muscle stages of *Trichinella spiralis*, as well as against larval stages of intestinal nematodes deeply embedded in tissue, such as *Trichuris* spp. [26,27]. Emodepside was also found to exhibit various antifilarial effects against different stages of *Acanthocheilonema viteae*, *Litomosoides sigmodontis* and *Brugia malayi* using the *Mastomys coucha* rodent model [28,29]. Furthermore, it was highly effective against *Onchocerca* spp. from cattle (adult *Onchocerca gutturosa in vitro* and Mf of *Onchocerca lienalis* in a surrogate mouse model), although it was less potent against *Brugia pahangi* [30].

In combination with praziquantel, emodepside was introduced into several veterinary markets across the world as Profender [26], the first topical dewormer for cats and as a tablet for dogs. One of the key characteristics of emodepside is that it remains fully efficacious against nematodes that are resistant to closantel, fenbantel, fenbendazole, levamisole and IVM in sheep and cattle, suggesting that cyclooctadepsipeptides exhibit a novel mechanism of action [31]. This was also confirmed in a multi-resistant hookworm isolate in dogs [32].

Latrophilins are classified as adhesion G protein-coupled receptors and latrophilin-1 is localised both pre- and post-synaptically in neurons [33]. *Lat-1*-deficient *Caenorhabditis elegans* was demonstrated to remain severely affected in locomotion and reproduction when exposed to emodepside, while showing a drastically reduced inhibition of the pharynx [34]. Subsequently, the large-conductance calcium-dependent, voltage-gated potassium channel SLO-1 was identified as essential for emodepside’s effects on the pharynx, motility and reproduction of *C*. *elegans* [35,36]. Using the *Xenopus laevis* oocyte expression system, it was demonstrated that emodepside directly opens the *C*. *elegans* SLO-1 channel [37]. Furthermore, an electrophysiological cross-phylum (nematode, arthropod and mammalian) comparison of the mode of action of emodepside indicated that its anthelmintic activity derives from strong and prolonged activation of nematode SLO-1 currents, not observed in the human channel [38]. Thus, based on its nematodicidal spectrum of activity [26], its strong activation of, and high affinity for, the nematode drug target SLO-1 over the mammalian ortholog [38,39], and moreover, its resistance-breaking properties [31,32], emodepside is considered to be one of the most promising developmental candidates for the treatment of human onchocerciasis and soil-transmitted helminthiases [40–43].

The identification of *slo-1* genes in all species across the phylum Nematoda gives hope for a broad anthelmintic for human use. However, characterisation of several splice variants and paralogues in some parasitic nematodes suggests that there are substantial differences in channel properties among species and lifecycle stages [37,44,45]. Here, we evaluate the macrofilaricidal activity of emodepside in the natural *O*. *ochengi*–cattle model.

## Materials and methods

### Ethics statement

The study design and the experimental procedures of the dose identification study in Germany were approved by the responsible authorities (LANUV–Landesamt für Natur, Umwelt und Verbraucherschutz NRW) under the reference number 200a166. All procedures performed on cattle in Cameroon were equivalent to those authorized by a Home Office Project License (Animals [Scientific Procedures] Act 1986) for related work on cattle in the UK. The study was also approved by the University of Liverpool’s Animal Welfare Ethical Review Board and a local, independent Animal Welfare Ethics Committee commissioned by the Cameroon Academy of Sciences.

### Identification of *slo-1* splice variants in *O*. *ochengi*

*O*. *ochengi* adult worms of both sexes were obtained from bovine skins purchased from Ngaoundéré abattoir, Adamawa Region, Cameroon. Viable, isolated worms were transferred into RNAlater (Ambion) to prevent RNA degradation and shipped on dry ice to the University of Liverpool where they were stored at –80°C. Worms were forwarded to Bayer, Germany, in RNAlater on gel-ice packs (4–8°C). Extraction of total RNA was performed using standard molecular biology procedures [46]. Briefly, total RNA was extracted from one entire male worm from which 1 μg of total RNA (DNase-treated) was reverse-transcribed to cDNA using a (dT)30 primer and SuperScript III Reverse Transcriptase (Invitrogen, Carlsbad, CA, USA). We used a basic local alignment search tool (BLAST) approach to identify the *slo-1* gene within the *O*. *ochengi* genome and designed forward and reverse primers to amplify the full-length open reading frame (ORF) by polymerase chain reaction (PCR). The gene was identified in both *O*. *ochengi* genome assemblies available on the WormBase ParaSite (PRJEB1465 and PRJEB1204) web platform [47,48]. Gene-specific primers located in the 3’ and 5’ untranslated region (UTR) of the gene were designed using Primer3 software [49]. The gene-specific PCR to obtain the full-length *Ooc-slo-1* from *O*. *ochengi* cDNA was performed using Phusion polymerase (New England Biolabs, Ipswich, MA, USA) and primer pair NheI_Ooc-slo-1_F1 (GGCGGCTAGCAGGAAGAAAAGATGAGCGATGTAT) and XhoI_Ooc-slo-1_R1 (GGCGCTCGAGGACTGGCACAACAAATCATAGAA). The reaction conditions were: 98°C for 60 s; 36 cycles of: 98°C for 15 s; 55°C for 30 s; 72°C for 3.5 min; and 72°C for 10 min. Corresponding ORFs were sequenced and cloned into the pT7-TS transcription vector (pBluescript, Agilent, Waldbronn, Germany) for subsequent *X*. *laevis* oocyte experiments. Sequence alignments of identified *O*. *ochengi slo-1* splice variants with *O*. *volvulus slo-1* splice variants (WormBase entry OVO4127 a–d, f) were performed using the default parameters of the Multiple Sequence Alignment tool MultAlin [50].

### Heterologous expression of *Onchocerca* spp. *slo-1* splice variants and assessment of emodepside sensitivity

Two identified *O*. *ochengi slo-1* splice variants and five *O*. *volvulus slo-1* splice variants were expressed in laboratory-bred *X*. *laevis* oocytes (EcoCyte Bioscience, Dortmund, Germany) for functional characterization. The cDNAs of five full-length *O*. *volvulus* splice variants were synthesized in accordance with available sequences predicted from the *O*. *volvulus* genome (WormBase entry OVO4127 *a*–*d*, *f*), while *O*. *ochengi* isoforms were amplified from cDNA (see above). The coding sequences (CDS) of each splice variant was cloned into the pT7-TS transcription vector (pBluescript) and integrity of the ORF was confirmed by Sanger sequencing. The pT7-TS vector is designed to introduce *X*. *laevis* beta-globin 5’ and 3’UTR to the insert sequence to enhance its expression in *X*. *laevis* oocytes. For all *slo-1* splice variants, capped cRNAs were synthesized (T7 mMessage mMachine kit, Ambion, Austin, TX, USA) from the linearized vectors containing the different *slo-1* splice variants according to the manufacturer’s protocol and cRNA samples were stored at –80°C. Oocytes were ordered from EcoCyte (Germany) and injected using standard procedures [51]. Oocytes were microinjected using a Roboinject automatic injection system (Multi Channel Systems, Reutlingen, Germany) with 30 nL of cRNA solution (250 ng/μL) and then incubated at 18°C in sterile-filtered Barth solution containing: NaCl (88 mM), KCl (1 mM), NaHCO_3_ (2.4 mM), HEPES (10 mM, pH 7.5), MgSO_4_·7H2O (0.82 mM), Ca(NO_3_)_2_·4H_2_O (0.33 mM) and CaCl_2_·6H2O (0.41 mM), at pH 7.4, and supplemented with 20 μg/mL of kanamycin, 100 U/mL penicillin, and 100 μg/mL streptomycin. Recordings were made 3–6 days post cRNA injection. Electrophysiological recordings such as current-voltage curves (IVCs) were performed using an automated two-electrode voltage-clamp AutoMate (HiClamp, multichannel system). Oocytes were impaled with two electrodes filled with 3 M KCl. To determine the effect of emodepside, cells expressing the different SLO-1 splice variants were clamped at –80 mV. Data were recorded at a frequency of 5,000 Hz during clamping, with the membrane potential between –60 and +80 mV for 500 ms using voltage steps of 20 mV. Between individual steps, the membrane potential was clamped to –80 mV for 1 s. All recordings were performed at 18°C ± 4°C and cells were superfused with OR2 medium containing: NaCl (88 mM), KCl (2.5 mM), HEPES (5 mM), MgCl_2_.6H_2_O (1 mM) and CaCl_2_.2H_2_O (1.8 mM) at pH 7.4. After initial recordings of IVCs, a perfusion with OR2 was performed for 1 min to determine the basal expression level of *Onchocerca* spp. SLO-1 in each cell. Next, five concentrations of emodepside (0.3, 1, 3, 10 and 30 μM in 0.1% dimethyl sulfoxide; DMSO) were tested on at least three cells per SLO-1 splice variant and IVCs measured after a 1 min incubation in the drug. The analysis was done using the HiClamp data acquisition and analysis software running under MATLAB (Mathworks Inc., Natick, MA, USA). Statistical analysis was performed using either Prism (GraphPad) or Excel (Microsoft). Plots of the peak outward currents at +20 mV (standardized to the current amplitude measured in OR2 buffer only) as a function of the logarithm of the drug concentration yielded classical concentration–activation curves and were readily fitted by single Hill equations. Concentration-activation curves were fitted with the equation:

$$\text{Y}=100/\left(1+10^{\text{H}(\text{logEC}50-\text{X})}\right)$$

where Y is the normalized response, logEC_50_ is the logarithm of the concentration of emodepside eliciting half-maximal current amplitude, X is the log of dose or concentration, and H is the slope factor or Hill slope.

### Bioanalytics of emodepside

Bovine samples (EDTA-plasma, skin snips and nodules) were stored at –80°C in Cameroon and shipped according to international transportation guidelines on dry ice to Bayer, Germany. Due to suspected contamination with other pathogens, samples required decontamination. Based on modified WHO guidelines for viral inactivation [52] and reports of virus stability in fluids and solid media issued by the United States Army [53], decontamination of samples was performed. Samples were thawed slowly in a microbiological safety cabinet and the plasma samples were vortexed gently. Skin snips and nodules were homogenized in a Qiagen TissueLyser II (Qiagen NV, Hilden, the Netherlands) with stainless steel beads. Homogenates were weighed and mixed with 4.5 volumes of a 0.5% collagenase solution (collagenase from *Clostridium histolyticum* [>125 IU/mg] Merck KGaA, Darmstadt, Germany), then incubated at 37°C with orbital mixing at 140 rpm for 48 h.

Plasma samples, skin and nodule homogenates were precipitated in a 1:10 ratio with acetonitrile using a vortex mixer at 150 rpm for 10 min, then centrifuged for 5 min at 14,000 rpm. The supernatants were pipetted into gas-tight vials and incubated at 80°C for 21 min to achieve a core temperature of 80°C for 10 min, then cooled to 4°C in a water-ice bath. Finally, samples were filtered using a cellulose sterile filter (S&S Spartan 13/30) into standard HPLC vials for liquid chromatography–mass spectrometry analysis. For liquid chromatography on a C18 column, a water/methanol gradient (containing 10 mmol ammonium acetate and 0.1 mL/L formic acid) was used. Quantification of emodepside was performed by tandem mass spectrometry on an API 5500 instrument (AB Sciex, Darmstadt, Germany) using the transition of m/z = 1,120 ([M + H]^+^) to its product ion m/z = 343. The extraction procedure was validated for plasma by concurrent recovery rates in the range 1–1,000 μg/L (*n* = 8, mean recovery 90%, relative standard deviation [SD] 5.4%) and for nodules and skin extracts between 1 and 1,000 ng/mL (*n* = 10, mean 105%, relative SD 16.2%).

### Dose identification in Holstein cattle

To ensure that doses of emodepside would be well tolerated in cattle in Cameroon, and that any adverse effects would be manageable in the field, dose identification was first performed in two female Holstein cattle (*Bos taurus*) 13–15 months old in Germany. Animals were from Rinder Union West, 48147 Münster, Germany, and were kept in a pen in an outdoor climate housing system of approximately 8 × 10 m in size in Hanscheider Hof, 51399 Burscheid, Germany. Pens were equipped with a self-locking feeding fence, deep-straw bedding and an automatic cup drinker system. Animals were offered fresh corn- and grass-silage once daily *ad libitum*, drinking water from the onsite well and mineral licks *ad libitum*. Straw bedding was refreshed at least weekly. Tolerability and pharmacokinetics (PK) of emodepside were assessed after a single intravenous (IV) injection: emodepside (4.0% m/m in solketal) was administered by constant infusion via the external jugular vein using disposable sterile IV catheters (Vasofix Braunüle 14G, 2,2 × 50 mm, Braun, Germany), sterile syringes (Perfusor-Syringe 50 mL, Braun, Germany), tubing systems (Braun Original-Perfusor-tubing, 150 cm, Luer Lock), and a perfusion system (Braun Perfusor Space). Animal 1 received emodepside 2.17 mg/kg; animal 2 received emodepside 1 mg/kg. Safety and tolerability were assessed during and after each administration. Animal 1 exhibited adverse effects and was stabilized with sedative (2 × 15 mg xylazine hydrochloride IV), corticosteroids (300 mg prednisolone-succinate-Na IV; 200 mg dexamethasone IV), glucose (750 mL glucose 5% IV), and saline (1.5 L sodium chloride 0.9% IV). To determine the PK profile of emodepside and any possible influence of the drug on blood glucose concentrations, blood samples were drawn before dosing and at 4 min, 8 min, 15 min, 30 min, 1 h, 2 h, 3 h, 4 h, 6 h, 8 h, 24 h, 32 h, 48 h, 72 h, 96 h and 168 h after administration. Samples for glucose determination were taken before dosing and at 15 min, 30 min, 1 h, 2 h, 3 h, 4 h, 6 h and 8 h after dosing, centrifuged at 10°C (3,220 × g) for 10 min and stored under cool conditions (<8°C).

### Modelling of single and multiple doses of emodepside for administration in cattle

Emodepside exposure in cattle after repeated IV administration was simulated based on two-compartmental evaluation of three PK profiles in zebu (Ngaoundéré Gudali) cattle (local breed; *Bos taurus indicus*): a single dose of 0.15 mg/kg, two doses of 0.15 mg/kg each 24 h apart, and a single dose of 0.75 mg/kg (75% of the maximum tolerated dose as a safety margin). In this study, we refer to the lower doses (0.15 mg/kg, singly or repeated) as the “humanized dose”. This is a plausible dose based on the human phase I trial data (in which single doses of up to 40 mg, and twice-daily doses of 10 mg for up to 10 days, were administered [54]). Modelling and simulations were performed using Phoenix WinNonlin (Certara) version 6.4 or later. A model using clearance and volume parameterization was produced to describe the concentration–time data. Model fitting and parameter estimation was performed with the Gauss–Newton algorithm and weighting with 1/(Yhat*Yhat). The obtained parameter estimates were used as fixed input for the simulations of PK profiles in cattle after seven daily IV administrations of 0.15 mg/kg or 0.75 mg/kg, respectively. Non-compartmental analysis of the simulated PK profiles was also performed to assess the impact of emodepside accumulation and to compare the efficacious exposure in cattle with the efficacious exposure in humans [54].

### Dose confirmation in zebu cattle with single-high, single-low and two consecutive low doses

The safety, tolerability and PK of low- and high-dose emodepside were confirmed in zebu (Ngaoundéré Gudali) cattle at the Veterinary Laboratory of L’Institut de Recherche Agricole pour le Développement (IRAD) at Wakwa (lat. 7°12’33”N, long. 13°34’20”E) near Ngaoundéré, Cameroon. Four non-pregnant, non-lactating cows naturally infected with *O*. *ochengi* were purchased from farmers in the Vina Division of the Adamawa Region and acclimatized at the IRAD Wakwa site for two weeks before the start of the trial. Cows were 3–6 years old and weighed 185–300 kg. They were housed in a 35 m^2^ enclosure at night and fed with cotton seed cake (2 g/kg bodyweight) in the morning before grazing them on a 1 ha *Brachiaria ruziziensis* paddock between 9 am and 4 pm daily. Water and salt were supplied *ad libitum* when housed. Each cow was randomly allocated (by ear-tag number using an online research randomizer [available at https://www.randomizer.org]) to receive a single low dose of emodepside 0.15 mg/kg, a single high dose of emodepside 0.75 mg/kg, a low dose twice (emodepside 0.15 mg/kg on two consecutive days), or vehicle only (solketal). Prior to treatment, the cows were sedated with xylazine hydrochloride (Rompun). Verum or vehicle was administered IV into the jugular vein using a perfusion apparatus (Braun Perfusor Space) following an identical protocol to the Holstein pre-study described above.

### Selection of cattle for evaluation of efficacy of emodepside against *O*. *ochengi*

Forty-two cows of the Ngaoundéré Gudali breed were purchased from local markets and farms where *O*. *ochengi* infection is endemic, across the Adamawa Plateau in northern Cameroon. Sample size was in part dictated by the upper limit of onsite facilities, a constraint typical of large-animal studies, and a group size of six (*n* = 7 here, permitting comparison of six dose or control groups) has been used effectively in previous drug studies in cattle [55,56]. Predetermined inclusion criteria were an *O*. *ochengi* nodule load of ≥21, weight 200–400 kg, and age 3–10 years (estimated by dentition). Exclusion criteria were evidence of pregnancy and/or lactation, poor body condition, suspected calcified *O*. *ochengi* nodules (abnormally hard on palpation), a positive skin reactivity test for bovine tuberculosis, dermal signs of infection with the bacterium *Dermatophilus congolensis* or heavy infestation with nodule-forming parasitic mites (*Demodex bovis*). After purchase, the animals were transferred to the premises of L’Institut de Recherche Agricole pour le Développement, Regional Centre of Wakwa, where transmission of *O*. *ochengi* is negligible. Health checks and routine vaccinations against endemic diseases were completed at least one month before the start of the experiment. Animals could drink from streams and lakes within a 2 km radius of sleeping pens, were free to graze by day and were confined at night. Salt was provided *ad libitum* and a cotton seed cake supplement was supplied at 200 g per 100 kg bodyweight during the study. Hay was provided during the dry season, supplemented with wheat and maize bran each morning before the animals went to graze. Herdsmen ensured animals did not enter zones of high parasite transmission.

Cattle were stratified by age, weight and dermal Mf load and randomly allocated (https://www.randomizer.org) to six treatment groups (seven animals/group) as previously described [56], such that the mean weight per group was 250–350 kg (group SD <50 kg) and the median age per group did not differ by ≥1 year. By group, the geometric mean Mf load per 100 mg skin ranged from 100 to 500 (Table 1); two animals found to be negative for skin Mf were distributed in different groups. Eighteen preselected nodules per animal were marked with tattoo ink, and their positions were recorded on a hardcopy ‘hide map’ and by digital video [57]. All assessments of drug efficacy were conducted by individuals blinded to the treatment groups. The study was conducted unmasked. All study personnel provided a signed declaration to conduct the study according to the specified protocol. Verum or vehicle was administered as described in the dose confirmation study.

**Table 1:** Characteristics of the treatment groups in cattle.

Group abbreviation	Treatment	Mean weight (± SD), kg	Median age (range), years	Geometric mean Mf load (SD factor), per 100 mg skin^#^	Animals lost during experiment (cause, time)
PCBO	Placebo (solketal), daily for 7 days*	290 ± 37	5 (3–10)	202 (17.8)	0
MRSM	Melarsomine, 4 mg/kg, every other day for 3 days	280 ± 33	5 (3–10)	335 (11.7)	0
EMO1L	Emodepside, 0.15 mg/kg, single dose	287 ± 32	5 (3–10)	278 (8.1)	0
EMO7L	Emodepside, 0.15 mg/kg, daily for 7 days	294 ± 40	5 (3–10)	122 (18.5)	2 (clostridial infection, day 21; stolen, day 60)
EMO1H	Emodepside, 0.75 mg/kg, single dose	309 ± 49	5 (3–10)	473 (6.0)	1 (stolen, day 90)
EMO7H	Emodepside, 0.75 mg/kg, daily for 7 days	289 ± 29	5 (3–10)	195 (19.7)	0

*Volume administered was equivalent to that in the EMO7H group.

^#^Geometric mean is based on a log_10_(×)+1 transformation to include zero counts. Baseline data were acquired at day 14.

Mf, microfilariae; SD, standard deviation.

### Measurement of blood glucose

Glucose concentrations were measured pre- and post-chemotherapy using venous blood, which was analyzed in a test-strip assay (MediTouch 2 blood glucose monitor, Medisana, Neuss, Germany). Both individual animal and group blood-glucose concentrations were compared with a standard reference range for cattle (45–75 mg/dL [58]). To evaluate group-level effects, 95% confidence intervals (CI) were calculated using the group mean, SD and sample number for each group at each respective timepoint, with changes in blood glucose concentration considered significant if either the lower CI exceeded the upper limit of the standard reference range, or the upper CI was below the lower limit of the standard reference range.

### Parasite enumeration and assessment of viability and fecundity

Tattooed nodules were removed under local anesthesia (lidocaine-epinephrine) on study days -1, 90, 180–190, 270–290, 360 and 550 in a randomized order as previously described [57]. Nodules were trimmed of excess tissue and up to four nodules per animal were processed separately as follows: (1) stored frozen at –80°C for PK analysis (up to day 14 only); (2) injected *in toto* with 10% neutral-buffered formalin and stored at 4°C for histopathology (up to day 290 only); or (3) dissected immediately in phosphate-buffered saline to separate adult male and female worms. The anterior end of the female worm (which even in viable specimens is the only motile part of the parasite) was removed from the worm mass and incubated alongside intact male worms for 30 min at 37°C for visual motility assessment on a three-point scale [59]. The remaining portion of the female worm mass was subjectively scored for fecundity on a four-point scale (0, uteri empty or worm structure degraded; 1, low; 2, intermediate; 3, normal) by examining the uterine contents microscopically *in situ*. This was followed by a fully quantitative embryogram to enumerate oocytes, embryonic stages and intrauterine Mf as described previously [59].

Worms from a second unfixed nodule (at the same timepoints listed above) were subjected to a 3-(4,5-dimethylthiazol-2-yl)-2,5-diphenyltetrazolium bromide (MTT) assay for metabolic activity [59], in which the insoluble formazan product was dissolved in DMSO and quantified colorimetrically at 620 nm in a microplate reader (model LT4500, Labtech, Heathfield, East Sussex, UK). Finally, dermal microfilarial densities were determined by incubation of skin biopsies (in triplicate per animal per timepoint) and normalized to 100 mg skin [59].

### Histopathology

Nodules for histopathology (*n* = 72) were collected up to day 290, after which few were available in the melarsomine (MRSM) group. For consistency, nodules were taken from the same three animals in each experimental group at each timepoint. Formalin-fixed nodules were embedded in paraffin, sectioned, and stained with hematoxylin and eosin or Giemsa as previously described [57,60] or with Lendrum’s carbol chromotrope [61] to specifically stain eosinophils.

Histological sections of nodules were scored by a single observer blinded to treatment group using the form proposed by Striebel [62], which was developed based on observations of *O*. *volvulus* (human) and *Onchocerca gibsoni* (bovine) nodules. In the current study, *O*. *ochengi* nodules were graded by applying a similar system in which the integrity of five structures or organs (cuticle, hypodermis, longitudinal muscle, pseudocoelomic cavity and gut) were classified through microscopical assessment of specific anatomical features (*e*.*g*., vacuoles in the hypodermis or host cells visible in the pseudocoelomic cavity) according to the categories in Table A in S1 Text. These grades were used to assign a pathology score for each aspect of parasite anatomy examined. Scores for all five structures or organs were then added together to produce a cumulative overall score, with a minimum score of 5 for parasite sections judged to be healthy across all five structures or organs. Since parasite sections showing extensive damage across all five anatomical regions could score a maximum of 15, sections containing cuticular debris only, or where parasites were unidentifiable in the section, were allocated a total score of 16. Where sections of male worms were present in the nodule these were scored separately to the females. Some sections contained more than one conjoined nodule. In these cases, females were also scored and recorded individually.

Cellular infiltrates within nodules, specifically polymorphonuclear cells (PMNs), were also assessed. Neutrophil and eosinophil counts were performed under oil immersion in cellular infiltrates immediately adjacent to sections of parasites on Giemsa-stained sections. A minimum of 200 cells were counted for each section and expressed as cells per high-power field. In sections containing no parasites, PMN counts were recorded if present in sufficient numbers. Histology sections stained by Lendrum’s method [61] were examined to quantify eosinophils. The PMNs were counted in cellular infiltrates immediately adjacent to sections of parasites as before (minimum of 100 differentiated cells/section).

The effect of different treatments relative to either (a) no treatment controls or (b) pre-treatment observations of parasite integrity was analyzed using linear mixed effects models with the nonlinear mixed effects model package in R [63,64]. Overall scores for parasite integrity were modelled as the response variable compared with (a) treatment groups for each timepoint and (b) different timepoints for each respective treatment group. In both cases, animal identity was modelled as a random effect to account for repeat observations taken from the same individual, and results were adjusted using a post-hoc Bonferroni correction for multiple comparisons. Similarly, to assess the effect of different treatments on cellular infiltrates over time, total PMN or eosinophil counts per high-power field and eosinophils as a percentage of the total PMN population were each modelled as explanatory variables compared with (a) treatment groups for each timepoint and (b) different timepoints for each respective treatment group.

### Pharmacokinetic–pharmacodynamic modelling of the effect of emodepside on *O*. *ochengi*

For the PK mean model, the PK of emodepside was modelled using a closed-form, two-compartment IV model according to Eq 1.

$${\text{c}}_{\text{sd}}\left(\text{t}\right)=\text{dose}*\text{c}0*\text{exp}\left(-\text{ke}*\text{t}\right)*\left(1+\text{delta}*\text{exp}\left(+\text{ke}*\text{f}*\text{t}\right)\right)$$ (1)

with ke, f and delta being parameters describing the distribution and elimination phase, and c0 being a proportionality factor for the dose.

The multiple IV dose was modelled as an additive single-dose, two-compartment model according to Eq 2.

$$c_{md}\left(t\right)=\sum_{i=1}^{7}t-i*t_{\Delta }>0?c_{sd}\left(t-i*t_{\Delta }\right):0$$ (2)

with i as index of doses (1 to 7), and t_Δ_ the difference in time between consecutive doses (here 24 h).

Parameters describing distribution and elimination of emodepside were fitted to the observed data of all dose steps and animals together using the optim fit function of R and the ‘L-BFGS-B’ method [65] without box constraints. From the observed data, the maximal observed concentration and the corresponding timepoint were extracted. The terminal half-life was calculated from the last three datapoints. The corresponding mean values were derived from the compartmental model parameters.

For pharmacokinetic/pharmacodynamic (PK/PD) modelling, we assumed a direct proportionality between drug concentration and drug effect according to Eq 3:

$$E=m*C+E_{0}$$ (3)

with E: PD endpoint, E_0_ the base (placebo) endpoint, m: a proportionality factor, and C the emodepside exposure.

Here, the null hypothesis, H0, is that the parameter m is not different to 0. If H0 can be rejected, a concentration-dependent effect of emodepside can be assumed. The alpha level selected for statistical significance was 5%. We considered three PD endpoints, a) uterine contents (with oocytes, developing embryonic stages, normal intrauterine Mf and degenerate intrauterine Mf considered separately), b) skin Mf load, and c) male and female motility (separately). Mf load is defined as the area under the curve of observed counts over time according to Eq 4:

$$Mfload=\sum_{i=2}^{n}\frac{Mfdensity_{i}+Mfdensity_{i-1}}{2}*\left(t_{i}-t_{i-1}\right)$$ (4)

per individual treated animal. To increase the robustness of the analysis, only animals presenting a skin Mf load of ≥100/100 mg skin at day -1 were included.

The same principle was applied to the analysis of the uterine contents. In order to test a dose-dependent effect, and an effect over placebo, the log load of uterine contents or Mf was used as the PD endpoint E in Eq 3, and exposure C was the sum of doses. Over all doses and individual animals, an effect size (m) and corresponding statistics were calculated using R’s lm function [66].

The second class of PD endpoint considered was worm motility, which was recorded as a three-class categorical variable and quantified here as a fraction of normal worm motility. First, a linear model of decrease in motility per dose group was established with a study-wide mean motility at day –1:

$$motility_{dose\_group,t}=mean\left(motility_{day-1}\right)-slope_{dose\_group}*t$$ (5)

Then, Eq 3 was applied for the PD endpoint ‘slope_dose_group_’ and the exposure as the sum of doses (performed separately for male and female worm motility).

All raw data underpinning this study are publicly available at Dryad (https://datadryad.org/stash) with the identifier doi:10.5061/dryad.vhhmgqns0 [67].

## Results

### Identification of two *slo-1* splice variants from *O*. *ochengi* adult worms

Using cDNA from *O*. *ochengi* samples, we were able to verify expression of the major emodepside target SLO-1 in *O*. *ochengi* adult male worms. In total, two different full-length CDSs of the *O*. *ochengi slo-1* gene, both 3,360 base pairs (bp) in length, were amplified and Sanger sequenced (splice variant a, GenBank: MW039265; splice variant b, GenBank: MW039266). The predicted amino acid sequences (1,119 amino acids) of both CDS were aligned against CDS of the *O*. *volvulus slo-1* gene, which has five predicted full-length splice variants (WormBase CDS OVO4127*a* –*d* and *f*) and one truncated splice variant (WormBase CDS OVO4127e) (Fig A in S1 Text). The two *O*. *ochengi* SLO-1 sequences shared the highest similarity with the *O*. *volvulus* SLO-1 splice variants *a* and *b*, respectively. The first *O*. *ochengi* sequence and *O*. *volvulus* SLO-1 splice variant *a* differ only in a single amino acid at position 658 (Arg to Lys) (Fig B in S1 Text). For the second identified *O*. *ochengi* sequence and its homologous *O*. *volvulus* splice variant *b*, two amino acid changes were identified at positions 658 (Arg to Lys) and 711 (Arg to Cys) (Fig C in S1 Text). The sequence alignments of the identified *O*. *ochengi* SLO-1 sequences with the corresponding *O*. *volvulus* splice variants revealed that SLO-1 is highly conserved between both species and that at least two SLO-1 splice variants are expressed in *O*. *ochengi* adult males.

### Functional expression of *Onchocerca* spp. isoforms in *Xenopus laevis* oocytes

**Fig 1:**
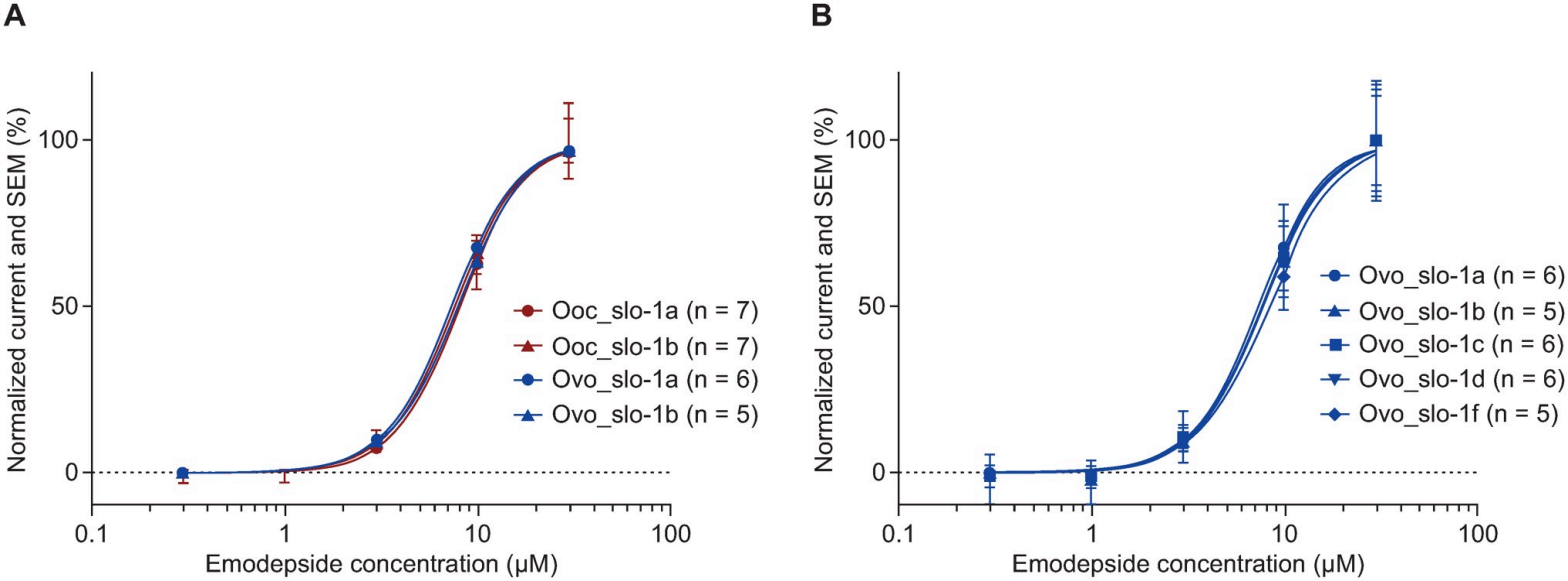
Emodepside sensitivity of *Onchocerca ochengi* and *Onchocerca volvulus* SLO-1 splice variants. *Onchocerca spp*. SLO-1 splice variants were heterologously expressed in *X*. *laevis* oocytes to determine species- and splice variant-specific effects of emodepside. All seven SLO-1 splice variants were able to form functional homomeric ion channels and changes in current could be measured with an electrophysiological two-electrode, voltage-clamp approach with oocytes exposed to increasing concentrations of emodepside (0.3, 1, 3, 10 and 30 μM). For each splice variant at each emodepside concentration, 5–7 oocytes were tested from a single pool of transfected cells to generate biological replicate data; the experiment was performed once. Datapoints are the mean values of the replicates at each emodepside concentration; error bars are standard error of the mean. Normalized concentration–response curves were fitted using the Hill equation. No statistical comparisons were made. Details of the DNA preparation, transfection, voltage clamp procedures and analyses are in the Materials and methods section. A Normalized concentration response curves for the two identified *O*. *ochengi* splice variants (red) and their two homologous *O*. *volvulus* splice variants a and b (blue). B Normalized concentration response curves for all *O*. *volvulus* full-length splice variants *a*–*d*, and *f*. Ooc_, *Onchocerca ochengi*; Ovo_, *Onchocerca volvulus*; SEM, standard error of the mean.

To investigate potential species- and isoform-specific differences in SLO-1 regarding emodepside sensitivity, we expressed the two identified *O*. *ochengi* splice variants and all five full-length *O*. *volvulus* splice variants heterologously in *X*. *laevis* oocytes to compare their responsiveness to emodepside. All heterologously expressed SLO-1 splice variants were able to form functional homomeric channels in the oocytes as shown by electrophysiological recordings. For SLO-1-expressing cells, an average baseline current of <3 μA was typically measured when cells were clamped at +20 mV under control conditions. When exposed to increasing concentrations of emodepside (0.3, 1, 3, 10 and 30 μM), no current increase effect was visible at concentrations below 3 μM. Maximal peak currents measured at +20 mV and 30 μM emodepside ranked from 19.1 μA for *O*. *volvulus* splice variant *f* to 39.9 μA for the *O*. *volvulus* SLO-1 splice variant *a*. Generated normalized concentration-response curves (Fig 1) revealed no notable differences in EC_50_ values (average EC_50_ ~7.8 μM) among any of the homomeric channels, indicating similar emodepside sensitivity of the drug target in both species and among all splice variants investigated (Table 2).

**Table 2:** Emodepside sensitivity of *Onchocerca ochengi* and *Onchocerca volvulus* SLO-1 splice variants. Two *O*. *ochengi* and five *O*. *volvulus* splice variants were heterologously expressed in *Xenopus laevis* oocytes and exposed to increasing concentrations of emodepside. Concentration-dependent changes in electric current were measured and concentration-response curves were generated. The EC_50_ value is the emodepside sensitivity of each SLO-1 splice variant.

Species	SLO-1 splice variant	Length (aa)	Emodepside sensitivity	
			EC_50_ (μM)	Standard error (μM)
*O*. *ochengi*	a	1,119	8.1	± 0.75
	b	1,119	7.7	± 0.52
*O*. *volvulus*	a	1,119	7.4	± 1.12
	b	1,119	7.8	± 1.04
	c	1,104	7.8	± 1.21
	d	1,104	7.8	± 1.20
	f	1,143	8.4	± 1.07

### Pharmacological effects of emodepside administration in cattle

Following perfusion in two Holstein cattle (perfusion rate: 45.2 mg/min; respiration 1.13 mL/min) of 2.17 mg/kg emodepside, treatment of the first animal needed to be discontinued due to severe generalised muscle tremor, salivation and tachycardia. As this animal was stabilised with glucose, blood samples for determination of glucose concentrations were not taken after perfusion and it recovered fully after 7 h. The second animal was perfused with a total dose of 1 mg/kg emodepside (perfusion rate: 22 mg/min; respiration 0.55 mL/min). During the application, its heart rate peaked at 140 beats per minute (bpm), then stabilised at approximately 108 bpm by the end of the perfusion. Salivation and slight muscle tremors were observed for approximately 1 h after application prior to full recovery of the animal. The perfusion of 1 mg/kg emodepside provoked a slowly increasing moderate hyperglycaemia, which peaked at ~12 mmol/L from 3–8 h post-application, with levels reverting to normal (4.33 mmol/L) after 24 h. Administration of xylazine for sedation at 6–8 h during skin biopsies may also have contributed to hyperglycaemia.

In the Cameroon pre-study in four zebu (Ngaoundéré Gudali) cattle, mild muscular tremors and salivation were observed over a period of 1 h following drug administration at the lower dose (0.15 mg/kg emodepside; perfusion rate, 0.6 mg/min or 0.07 mL/min). However, no salivation and only very transient muscular tremors were apparent after a second low-dose (0.15 mg/kg) treatment without xylazine sedation. Heart rate was mildly affected by the high-dose (0.75 mg/kg) treatment, increasing from 72 to 80 bpm at 6 h post treatment, which was maintained over the next three days before returning to normal on day 4. Rumen contraction increased from 1 contraction/min with both the low-dose and high-dose treatments to 1.5 and 2 contractions/min, respectively, over the next four days after treatment. Temperature and respiratory rates were not affected. High-dose treatment provoked a rise in blood glucose concentration to 158–200 mg/dL 8 h after treatment, which dropped to 61 mg/dL (within the normal range) by 24 h. A similar rise in glucose level was observed with the low-dose treatments.

### Modelling of single and multiple humanized or high doses of emodepside for administration in cattle

The PK profile of emodepside in plasma samples after administration to Holstein cattle was obtained via the jugular vein at concertinaed intervals from 4 min to 168 h. The two-compartmental PK model predicted an initial plasma concentration of 120 μg/L in the low-dose (“humanized”) group (0.15 mg/kg) and ~2,400 μg/L in the high-dose group (0.75 mg/kg) (Fig D[a] in S1 Text). Double treatment of low-dose emodepside at 24-h intervals was predicted to ensure that a minimal plasma level of 5 μg/L (reached with the single low-dose treatment at 24 h) would be maintained for up to 72 h (Fig D[b] in S1 Text). Simulations of seven consecutive daily doses of emodepside were generated based on the obtained PK parameters for 0.15 mg/kg and 0.75 mg/kg. The concentration–time profiles (Fig D[a] in S1 Text) and non-compartmental analysis results (Table B in S1 Text) predicted no relevant accumulation of the peak concentration (C_max_) and only moderate accumulation of the area under the curve (AUC) between day 1 and the steady state of daily emodepside treatment. Based on these observations, the following low- and high-dose emodepside regimens and controls were selected for comparison in the main trial: a single dose of emodepside 0.15 mg/kg (EMO1L), or of emodepside 0.15 mg/kg daily for seven days (EMO7L); a single dose of emodepside 0.75 mg/kg (EMO1H), or of emodepside 0.75 mg/kg daily for seven days (EMO7H); a placebo control (PCBO) of solketal daily for seven days; and a positive control of MRSM 4 mg/kg every other day for three days.

### Emodepside distribution in plasma and skin following single and multiple humanized or high doses

**Fig 2:**
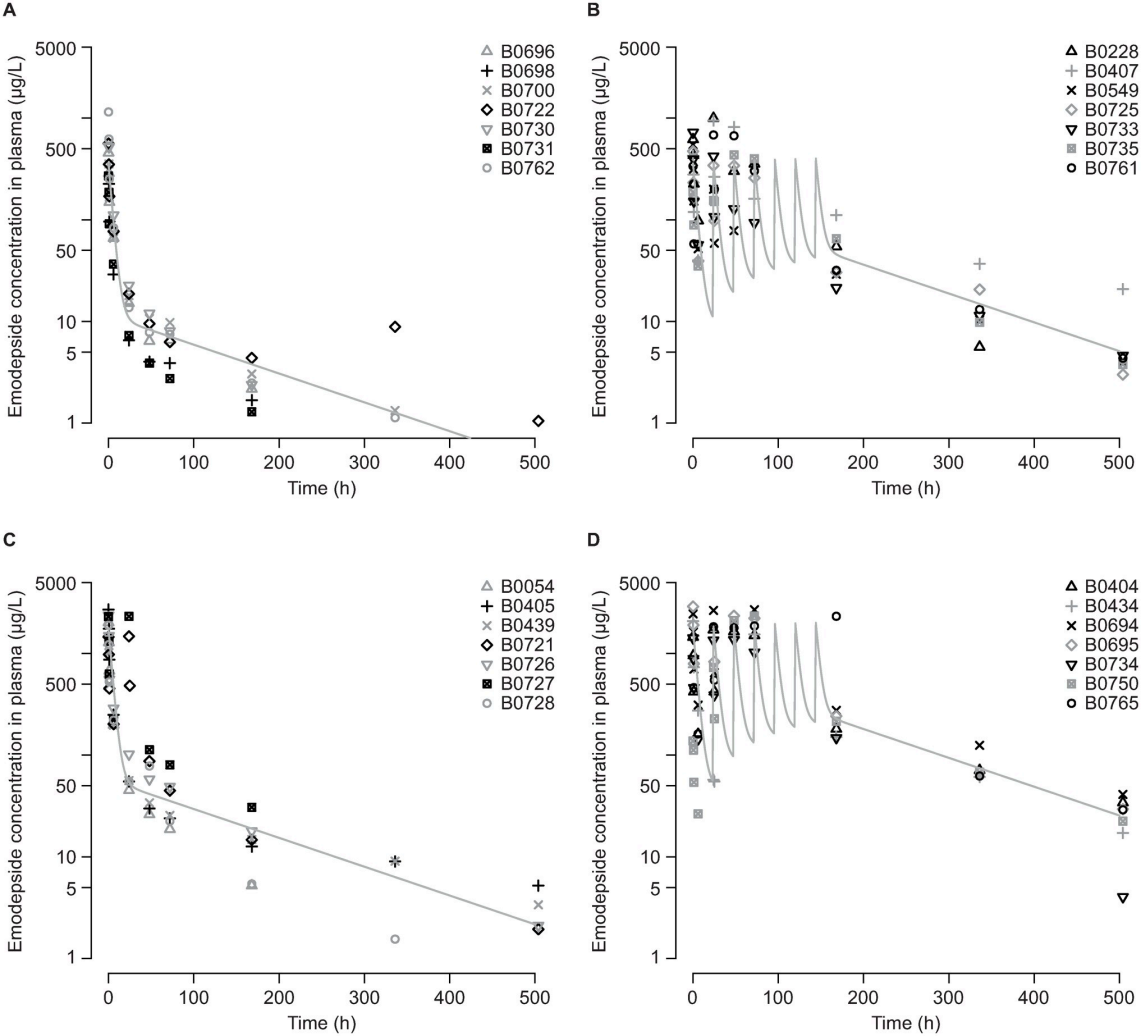
Observed plasma PK (concentration vs time curves) for four emodepside dose steps in zebu (Ngaoundéré Gudali) cattle (*B*. *t*. *indicus*). Dose groups were intravenous emodepside: A 0.15 mg/kg/day on day 0. B 0.15 mg/kg/day on days 0–6. C 0.75 mg/kg/day on day 0. D 0.75 mg/kg/day on days 0–6. Emodepside concentration in plasma was determined by tandem mass spectrometry using serial dilutions of single samples per animal (*n* = 7 per dose group) at each timepoint. Symbols represent observations for individual animals; the lines represent the PK model of emodepside for that dosing regimen. Data are from a single study. No statistical comparisons were made.

In the main trial in Cameroon, it was prespecified that after inclusion, animals could only be excluded from the study on health grounds. None were excluded but three animals were lost to the experiment for reasons unrelated to the drug treatments (Table 1). As this occurred after the treatment regimens had been completed, data for these animals were included in the PK analysis and the PD analysis for dermal Mf load. A two-compartment model was fitted to the observed plasma concentrations of emodepside; AUC, terminal half-life (t_½_), C_max_, and the time taken to reach C_max_ (T_max_) were derived from the PK model then compared with the observed C_max_ and T_max_ (Table C in S1 Text). In the single dose groups (EMO1L [0.15 mg/kg–“humanized”] and EMO1H [0.75 mg/kg]), the observed C_max_ was usually apparent from the first sampling (5 min). The terminal half-life was derived from observed data and aggregated through the PK model to approximately 106 h. Fig 2 displays the observed plasma concentration at different dose steps for individual animals and the respective mean PK model. The emodepside concentration in the skin varied substantially. On 54 occasions, skin and plasma concentrations were measured on the same study day. Although the median ratio between skin and plasma was approximately 1.1, it ranged from 0.03 to 93 (Fig E in S1 Text).

### Effects of emodepside on blood glucose

**Fig 3:**
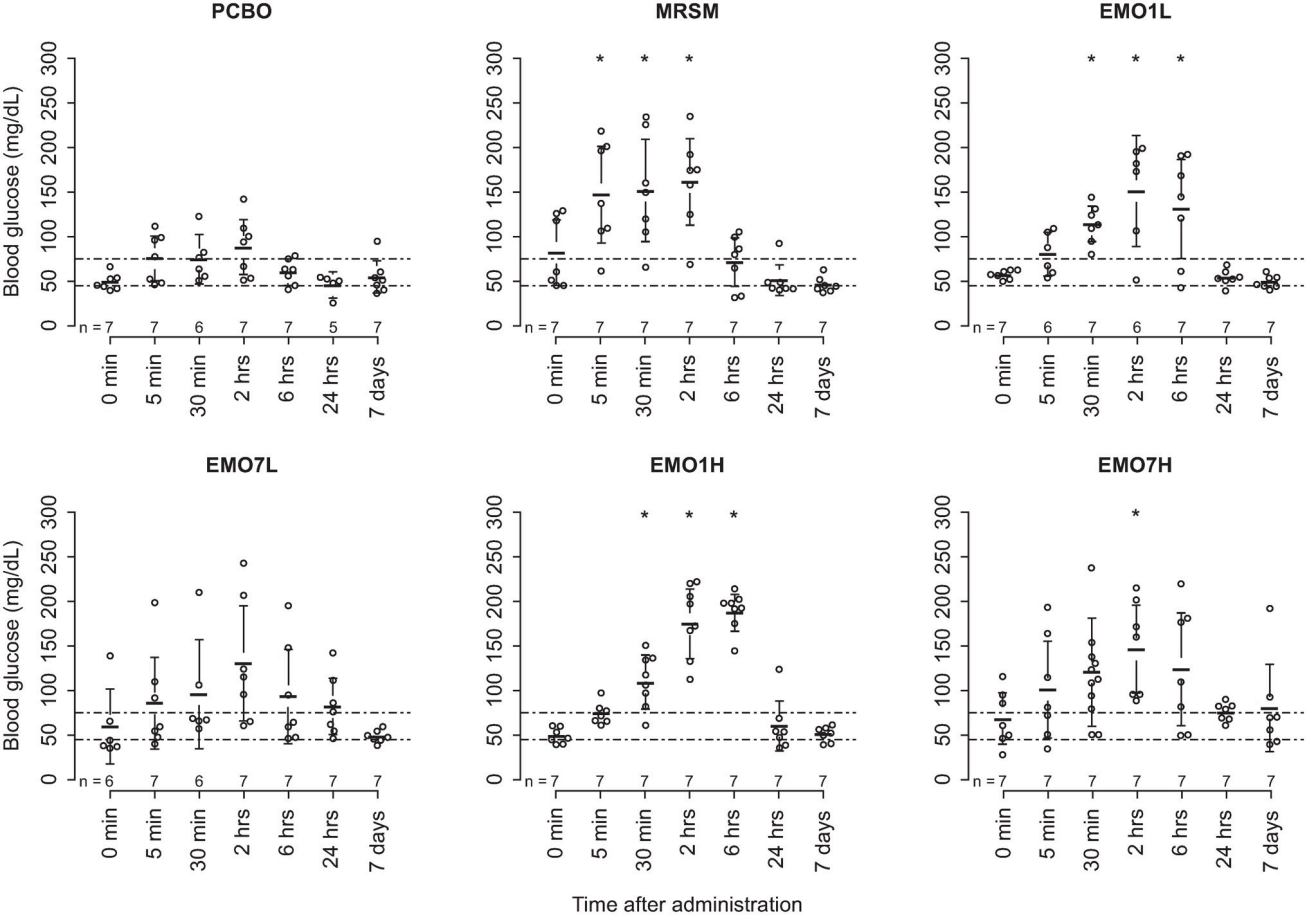
Blood glucose concentrations observed pre- and post-treatment commencement with emodepside by experimental group in zebu (Ngaoundéré Gudali) cattle *(B*. *t*. *indicus)*. Glucose concentration in venous blood was determined by test strip assay (MediTouch 2 blood glucose monitor, Medisana, Neuss, Germany). Treatment groups were single dose, or seven daily doses, of emodepside at 0.15 mg/kg (EMO1L, EMO7L) or 0.75 mg/kg (EMO1H, EMO7H), placebo (PCBO [solketal]) daily for seven days, and melarsomine (MRSM) 4 mg/kg every other day for three days as a positive control. Points represent single observations in individual animals. Bars and whiskers represent group means with 95% CI. Normal distribution was assumed because of the even distribution of individual blood glucose values above and below each group mean at each timepoint. The broken horizontal line represents the standard reference range for bovine blood glucose concentrations. Timepoints where the group-level blood glucose 95% CI was elevated above this range are marked with an asterisk. No formal statistical comparisons were made. EMO1H, emodepside, 0.75 mg/kg, single dose; EMO7H, emodepside, 0.75 mg/kg, daily for seven days; EMO1L, emodepside, 0.15 mg/kg, single dose; EMO7L, emodepside, 0.15 mg/kg, daily for seven days; MRSM, melarsomine, 4 mg/kg, every other day for three days; PCBO, placebo.

Observed changes in blood glucose are summarised in Fig 3. Concentrations for individual animals varied considerably between individuals, groups, and over time. Increases in serum concentration following commencement of treatment were observed in animals in all experimental groups for 6–24 h before returning to baseline levels. These increases were significantly above the standard reference range for cattle (45–75 mg/dL [58]) at the group level following MRSM treatment at 5 and 30 min and 2 h; both low- and high-dose single emodepside groups (EMO1L and EMO1H, respectively) at 30 min, 2 and 6 h; and in the high-dose multiple emodepside group (EMO7H) at 2 h following treatment commencement (Fig 3).

### Effects of emodepside on microfilarial density and embryogenesis

**Fig 4:**
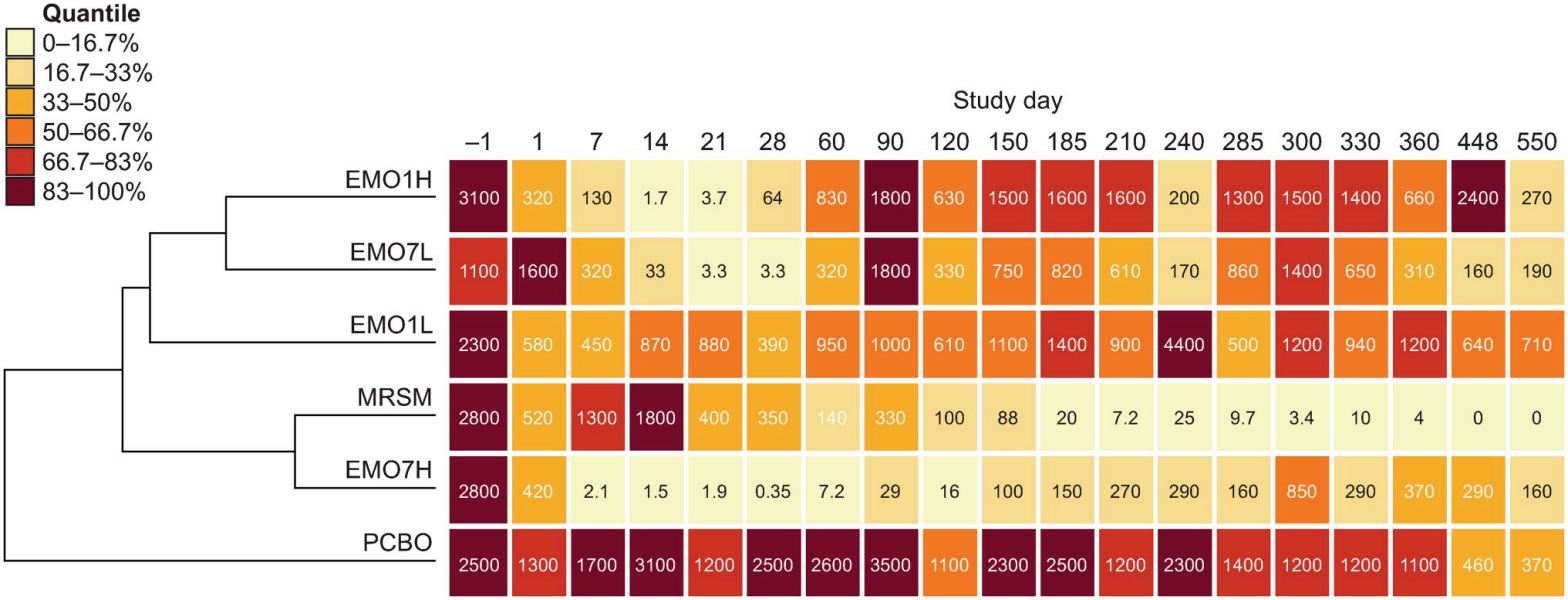
Mean dermal microfilarial density (*n* per 100 mg skin) in zebu (Ngaoundéré Gudali) cattle (*B*. *t*. *indicus*) treated with emodepside or melarsomine compared with placebo. Mean dermal microfilarial density was determined by incubation of skin biopsies (in triplicate per animal, per timepoint) normalized to 100 mg skin [59]. Mean density values are shown at each timepoint in each treatment group (*n* = 7 per group) and are color-coded by quantile, from pale yellow (low microfilarial density) to dark red (high microfilarial density). The dendrogram was created by hierarchical clustering based on Euclidean distance between mean dermal microfilarial density profiles and indicates the degree of similarity between treatments based on the *in vivo* response over time. No between-group statistical comparisons were made. EMO1H, emodepside, 0.75 mg/kg, single dose; EMO7H, emodepside, 0.75 mg/kg, daily for seven days; EMO1L, emodepside, 0.15 mg/kg, single dose; EMO7L, emodepside, 0.15 mg/kg, daily for seven days; MRSM, melarsomine, 4 mg/kg, every other day for three days; PCBO, placebo.

Sampling of skin Mf during chemotherapy was concertinaed to ensure that early effects of emodepside (*i*.*e*., within the first three months) on this parasite stage were captured in detail (Fig F in S1 Text). Mean Mf levels were reduced by 10–1,000-fold in the high-dose groups (EMO1H and EMO7H) within seven days, whereas EMO7L took 14 days to reach a > 10-fold decrease and EMO1L never attained a reduction of this magnitude (Fig 4). For placebo (PCBO), mean Mf densities remained above 1,000/100 mg skin until year 2, after which they declined to ~400 Mf/100 mg skin. Despite this natural reduction in Mf levels in the absence of treatment, hierarchical clustering analysis placed PCBO as an outgroup and clustered MRSM and EMO7H together (Fig 4); these two groups exhibiting the most sustained declines in Mf densities. However, the kinetics of Mf reduction showed important differences between these two groups, with a slow but consistent decline in MRSM leading to complete clearance after one year, compared with a sharp initial effect of chemotherapy in the first few weeks followed by a partial recrudescence from 10 months with EMO7H (Fig 4). Although recoveries approaching pre-treatment levels were observed as early as day 90 in EMO7L and EMO1H, the reductions compared with baseline at 12 months for EMO1H and EMO7H were 79% and 87%, respectively, and by 18 months had reached 91% and 94%, respectively.

The effects of emodepside on oocyte numbers were apparent at day 90 with all regimens, with recoveries to PCBO levels at day 550 in the single-dose groups contrasting with extirpation of oocytes in the multiple-dose groups (Fig G[a] in S1 Text). A clear impact on developing embryonic stages was also observed at day 90 in the multiple-dose groups and they were virtually eliminated by these regimens by day 550 despite an intervening period of recovery (Fig G [b] in S1 Text). The pattern for intrauterine Mf largely mirrored the effects seen on the earlier stages, in that the multiple-dose groups cleared normal Mf from the uteri by day 550, unlike the single-dose regimens (Fig G[c] in S1 Text). In the high-dose groups, numbers of degenerated Mf increased earlier (at day 90) than with the low-dose regimens (at day 185) (Fig G[d] in S1 Text). Overall, the embryotoxic effects of emodepside were observed much later in the experiment than for the positive control (MRSM).

### Effects of emodepside on adult worm fecundity, viability and nodular infiltrates

Female worm motility was reduced to zero by day 90 in all nodules recovered from MRSM (Fig H[a] in S1 Text). In contrast, no dose of emodepside achieved this level of motility inhibition, although female worms recovered from 6/7 animals in the EMO7H group displayed motility scores of 0 at day 550 (Fig H[b] in S1 Text). At the same timepoint, differences in MTT reduction between MRSM and EMO7H were more marked; while no intact worms were recovered from the former (Fig I[a] in S1 Text), female worms assayed from 4/7 animals in EMO7H exhibited normal metabolism (Fig I[b] in S1 Text). However, female fecundity was profoundly affected by all doses of emodepside by the end of the experiment, with EMO1L, EMO7L and EMO7H displaying scores of <0.2 at day 550 (Fig J in S1 Text). Effects of emodepside on the motility of adult male worms appeared to be more limited than for females (Fig K in S1 Text).

**Fig 5:**
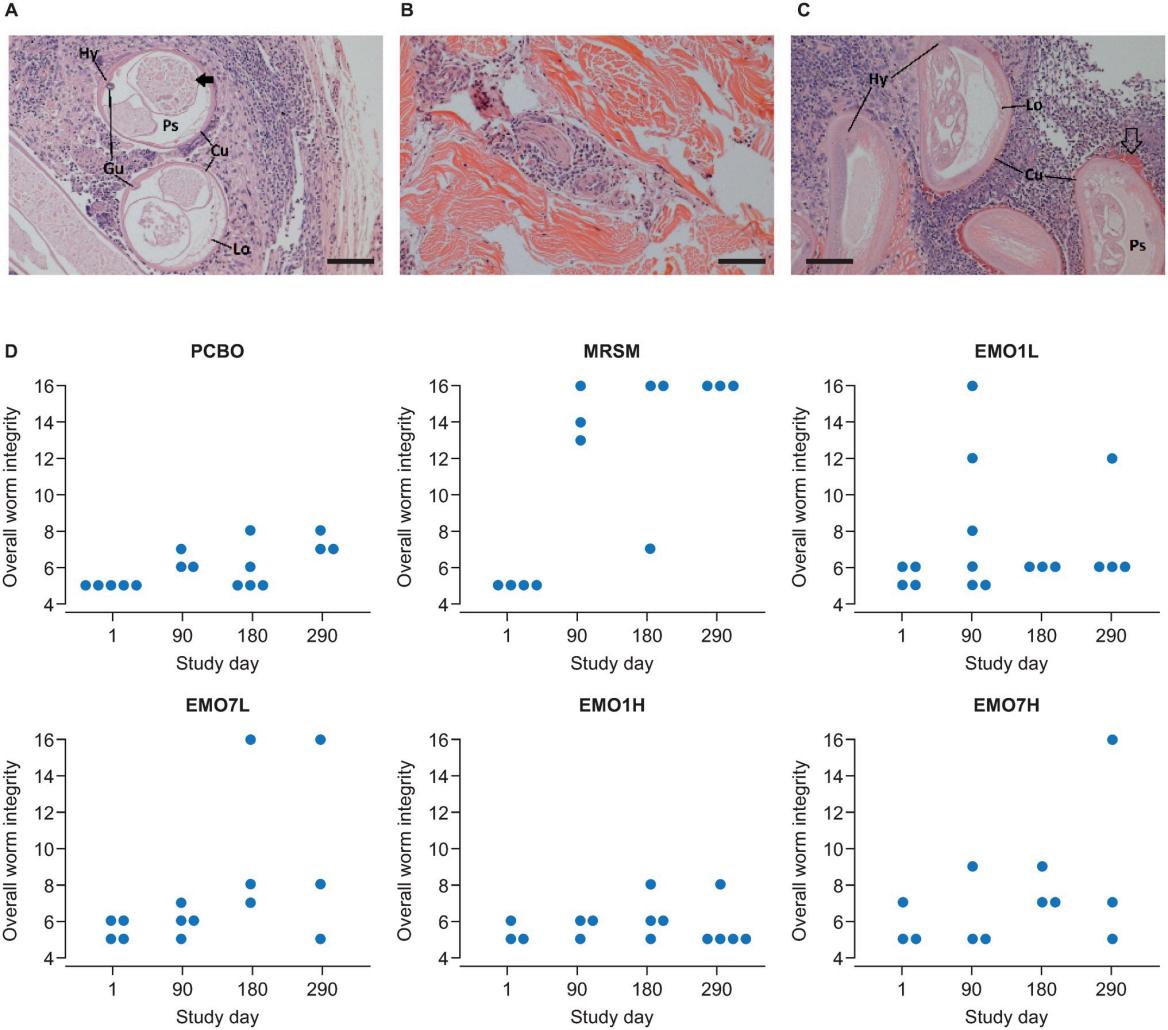
Histopathology of *Onchocerca ochengi* nodules treated with emodepside. Parasite sections were scored according to Striebel [62]. Scoring was based on the overall condition of cuticle (Cu), hypodermis (Hy), longitudinal muscle (Lo), pseudocoelomic cavity (Ps), and gut (Gu; when present in section). Age, reproductive status and nodular environment were also assessed. Micrographs (scale bars, 100 μm) show representative sections for experimental groups at day 290 for: A Placebo (PCBO), showing transverse and oblique sections of a viable (animal ID B0406; score 7), non-productive female. Solid arrow denotes debris within the uterine body. B Melarsomine (MRSM), showing a degenerate nodule composed predominantly of collagen with foci of perivascular fibroblasts and polymorphonuclear cells. No evidence of parasites is present within the section (animal ID B0435; score 16). C High-dose emodepside (EMO7H), showing multiple transverse sections of a viable (animal ID B0734, score 7), non-productive female in a nodule with moderate polymorphonuclear cell infiltrate. Open arrow denotes eosinophilic Splendore–Hoeppli deposits. D Plots show cumulative histopathological scores (across worm tissues and organs) by experimental group and timepoint (see Materials and methods for scoring procedure). Datapoints are from a single nodule from different animals at each timepoint and include scores for male worms, where observed in nodules (Table D in S1 Text). EMO1H, emodepside, 0.75 mg/kg, single dose; EMO7H, emodepside, 0.75 mg/kg, daily for seven days; EMO1L, emodepside, 0.15 mg/kg, single dose; EMO7L, emodepside, 0.15 mg/kg, daily for seven days; MRSM, melarsomine, 4 mg/kg, every other day for three days; PCBO, placebo.

Histopathological analysis of nodules supported the direct observations of the adult parasites. A total of 72 nodules were taken from 18 animals across four timepoints (up until day 290) for histological assessment, yielding 77 parasite sections (62 females and 15 males; Table D in S1 Text). Twelve sections contained either cuticular debris only or no identifiable parasites (*e*.*g*., Fig 5B), including two nodules removed pre-treatment from animals in MRSM and EMO1H, respectively. Data for these two nodules were excluded from subsequent analyses. Overall, the analysis of parasite integrity based on cumulative scores indicated that worm sections present in MRSM nodules had significantly higher pathology scores than PCBO (*P* <0.05) at all post-treatment timepoints (Tables D and E in S1 Text; Fig 5). Scores also increased significantly in MRSM worm sections for all post-treatment timepoints when compared with pre-treatment scores within this group (Fig 5). However, no significant effect of emodepside on worm integrity across the four dosing groups was apparent (Fig L and Table F in S1 Text).

Regarding host cell infiltrates within nodules, PMN numbers increased in EMO7L and EMO1H over time, although statistical significance was not achieved in either case (Fig M and Table G in S1 Text). No such trend was observed in EMO7H, although there were confounding effects of exceptionally high pre-treatment PMN counts in this group. In EMO7L and EMO1H, the proportion of eosinophils within the intranodular PMN populations decreased significantly over time (Fig N and Table H in S1 Text).

### PD modelling of the effect of emodepside on *O*. *ochengi*

**Fig 6:**
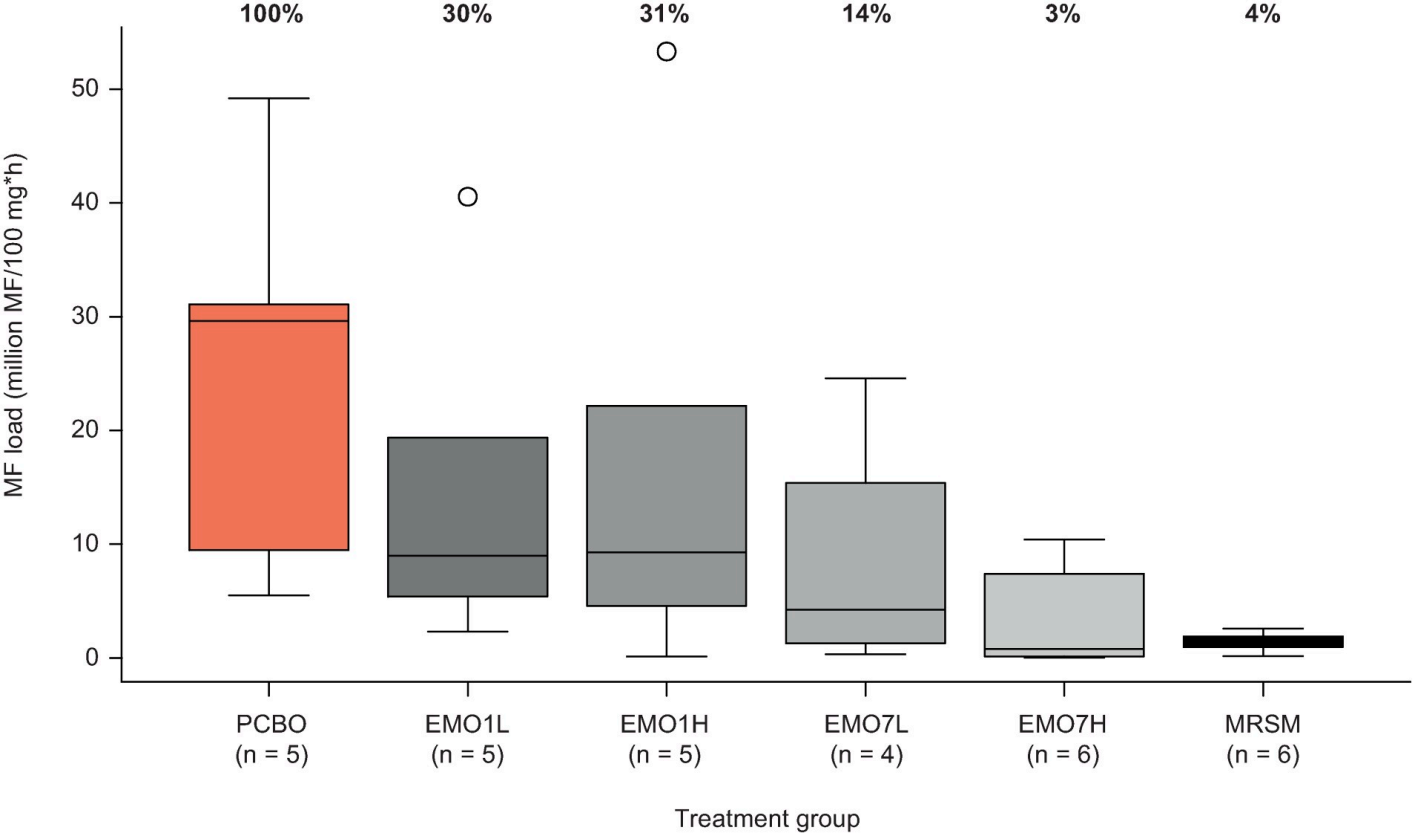
Dermal microfilarial load distribution (area under the curve [load*time]) by treatment group in zebu (Ngaoundéré Gudali) cattle (*B*. *t*. *indicus*). Dermal microfilarial density was determined at each timepoint by incubation of skin biopsies (in triplicate per animal at each timepoint), normalized to 100 mg skin [59], then used to estimate total area under the load*time curve. The boxplot is ordered by dose steps of emodepside (grey shading). The thick bar is the median, the limits of each box are the interquartile range, the whiskers are the smallest/largest value within 1.5× the interquartile range, and the open circles are outliers. The placebo control and melarsomine (positive control) are shown in orange and black, respectively. Median microfilaria load per treatment group as a proportion of placebo (normalized to 100%) is shown above the plot. No between-group statistical comparisons were made. EMO1H, emodepside, 0.75 mg/kg, single dose; EMO7H, emodepside, 0.75 mg/kg, daily for seven days; EMO1L, emodepside, 0.15 mg/kg, single dose; EMO7L, emodepside, 0.15 mg/kg, daily for seven days; MF, microfilariae; MRSM, melarsomine, 4 mg/kg, every other day for three days; PCBO, placebo.

**Fig 7:**
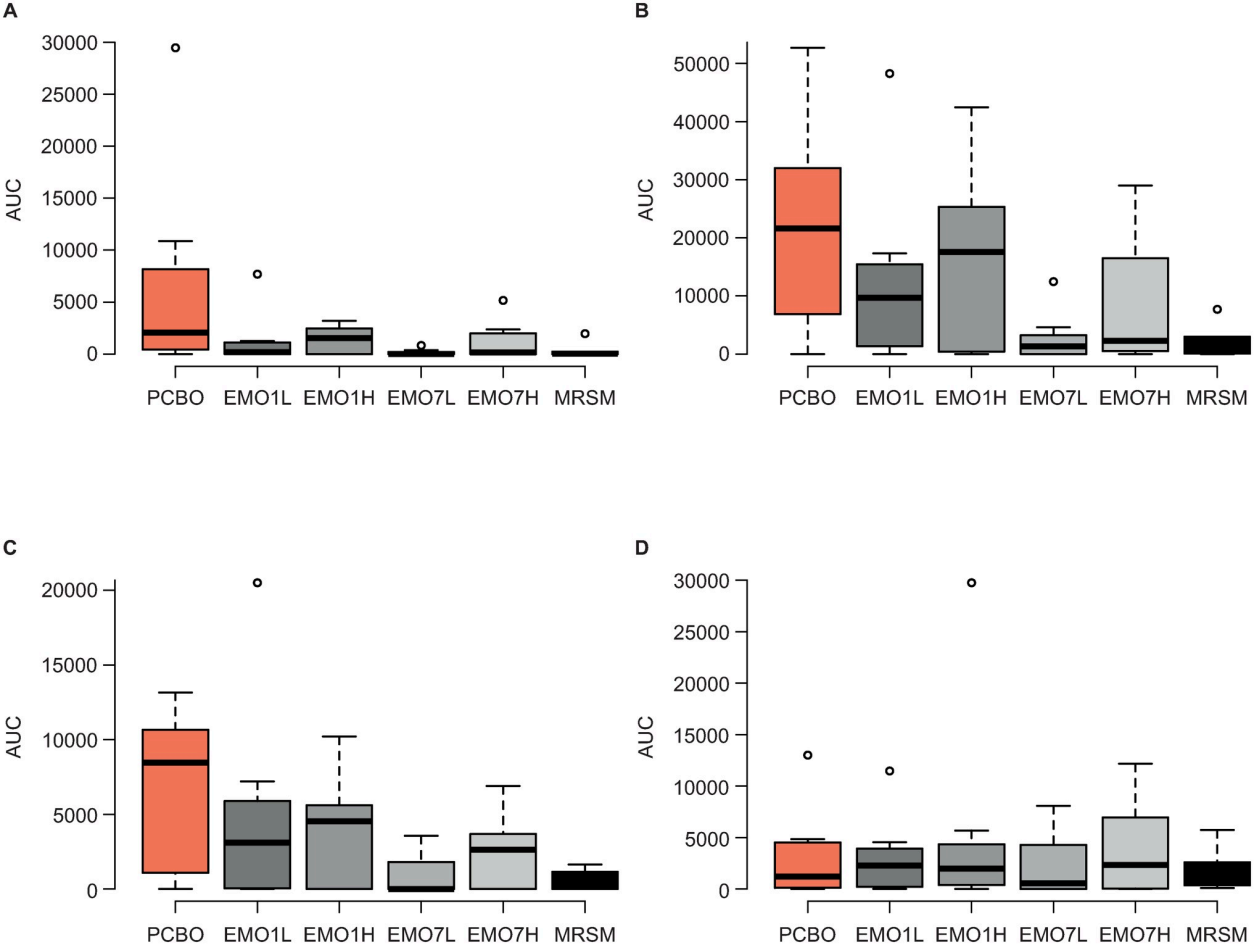
Distribution of counts for uterine contents at different dose steps. (A) oocytes, (B) all developing embryonic stages, (C) normal, and (D) degenerated intrauterine microfilariae in cattle treated with emodepside or melarsomine compared with placebo. Counts for uterine contents were determined in samples from each animal (*n* = 7 animals per treatment group), then used to estimate total area under the load*time curve. The boxplot is ordered by dose steps of emodepside (grey shading). The thick bar is the median, the limits of each box are the interquartile range, the whiskers are the smallest/largest value within 1.5x the interquartile range, and the open circles are outliers. The placebo control and melarsomine (positive control) are shown in orange and black, respectively. EMO1H, emodepside, 0.75 mg/kg, single dose; EMO7H, emodepside, 0.75 mg/kg, daily for seven days; EMO1L, emodepside, 0.15 mg/kg, single dose; EMO7L, emodepside, 0.15 mg/kg, daily for seven days; MRSM, melarsomine, 4 mg/kg, every other day for three days; PCBO, placebo.

We tested the hypothesis that emodepside had dose-dependent effects on dermal Mf density and adult worm motility. The variability of the data required a robust approach to aggregate individual observations. Therefore, we calculated the integral of the curve that describes Mf density over time, denoted here as the Mf load. In comparison with MRSM, the emodepside groups displayed higher variability in Mf load, although EMO7H achieved a similar median reduction (97%) to MRSM (96%) (Fig 6). Notably, the single-dose groups (EMO1L and EMO1H) exhibited reductions in Mf load of only ~70%, whereas EMO7L attained a reduction of 86% (Fig 6). To analyse the relationship between Mf load and emodepside dose, a linear model of the sum of drug doses and log observed Mf load was developed, which demonstrated a significant negative association between these variables (*P* = 0.0042). Surprisingly, applying the same approach to the impact of emodepside on counts for uterine contents (oocytes, developing embryonic stages and intrauterine Mf) did not reveal a significant overall dose-dependent effect. However, the AUC for all embryonic stages was significantly reduced in the EMO7L group (*P* = 0.048 compared with *P* = 0.005 for MRSM) (Fig 7).

**Fig 8:**
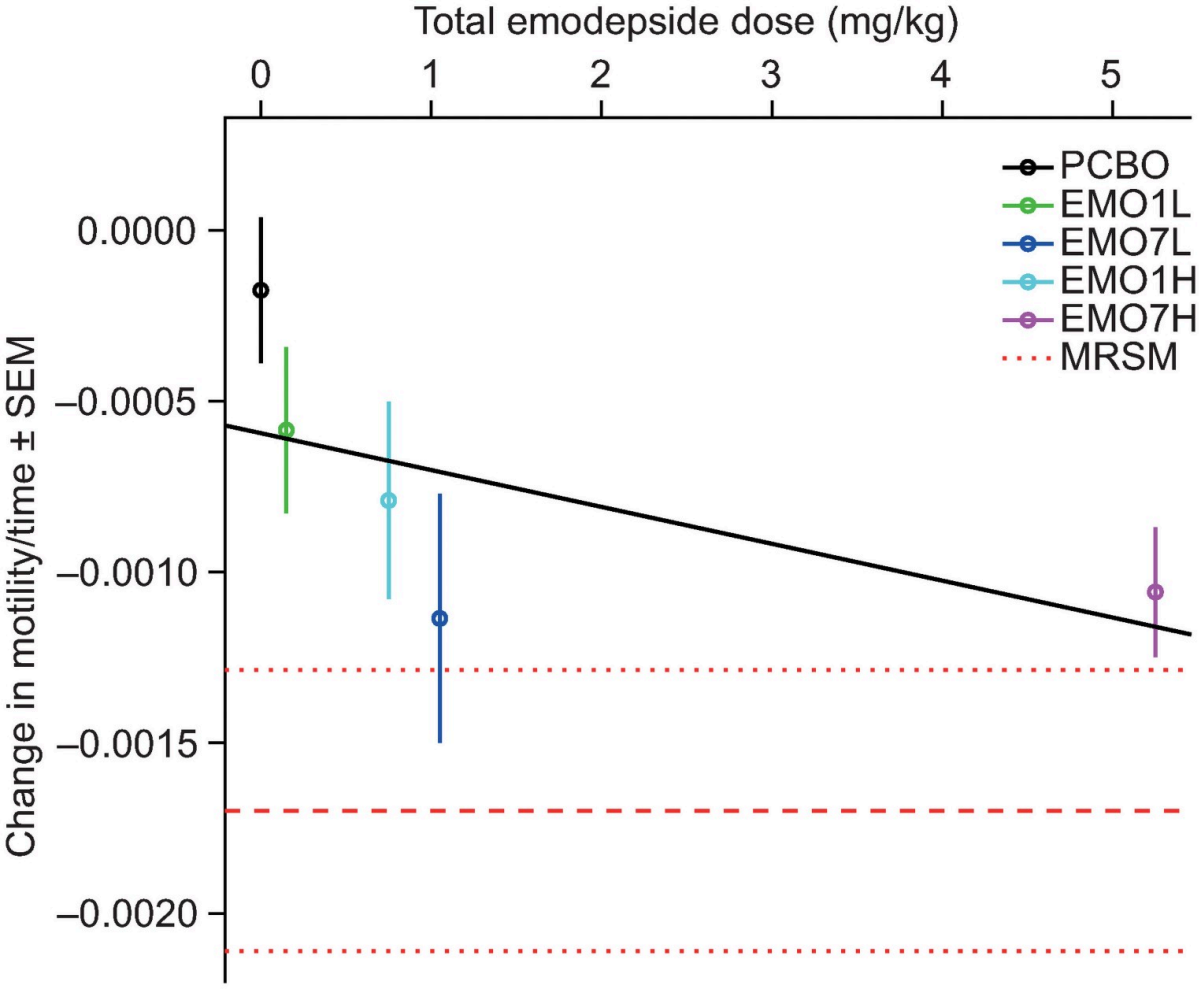
Linear model describing the relationship between change in adult female worm (*Onchocerca ochengi*) motility over time and total emodepside dose. The anterior end of the female worm was removed from the worm mass and incubated alongside intact male worms for 30 min at 37°C for visual motility assessment on a three-point scale [59]. A linear model of worm motility over time for a given treatment group was estimated and repeated for each group; the slope β for each group is reported in this figure. The null hypothesis was β = 0, and *t* statistics were used to test and reject the null hypothesis and to estimate the *P* value. Datapoints are mean values and error bars represent SEM. Dashed lines in red indicate the mean ± SEM for the positive control (MRSM). A regression model of the relationship between dose and decrease in motility (black line) indicated there was a non-significant trend between dose and treatment effect. EMO1H, emodepside, 0.75 mg/kg, single dose; EMO7H, emodepside, 0.75 mg/kg, daily for seven days; EMO1L, emodepside, 0.15 mg/kg, single dose; EMO7L, emodepside, 0.15 mg/kg, daily for seven days; MRSM, melarsomine, 4 mg/kg, every other day for three days; PCBO, placebo; SEM, standard error of the mean.

Adult female worm motility was highly variable across the six treatment groups at day −1, ranging from 85% to 43% (overall mean 64%; Table I in S1 Text). Moreover, in PCBO over time, the lowest recorded motility was 40% (at day 275). This variation reflects the natural range of nodule (and therefore worm) ages between animals, with some nodules containing senescent individuals. Therefore, a linear model was built on the assumption that the initial motility value was 64% across all groups and decreases linearly over time. The model clearly demonstrated that the slope (decrease of motility over time) is significant (*P* <0.05) for all groups except PCBO, and the effect increased with dose (Fig 8). Male worm motility on day –1 was uniformly high (classified as normal in all cases), while post-treatment data were missing in MRSM, presumably due to the rapid macrofilaricidal activity of melarsomine on male worms migrating between nodules (Table I in S1 Text). The onset of a reduction in male motility was late in the emodepside groups (day 360) and only EMO7H showed a significant decrease (Table I in S1 Text). While the reduction in male motility was not significant in the other emodepside groups individually, the decrease was significantly related to dose (*P* = 0.003); *i*.*e*., a higher overall dose results in a greater decrease in male motility (Fig O in S1 Text).

## Discussion

Here, we demonstrate that emodepside rapidly reduces the density of skin Mf, suppresses female fecundity for at least 18 months, and (after multiple applications of a high dose) induces partial macrofilaricidal effects, predominantly against females. To maximise the predictive power of this preclinical study for assessing the efficacy of emodepside against human onchocerciasis, three key steps were taken to validate the bovine screening system. First, the pharmacological sensitivity of SLO-1 isoforms from the cattle parasite, *O*. *ochengi*, was demonstrated to be equivalent to that of the human parasite, *O*. *volvulus*. Second, modelling of single and multiple humanized doses was performed using the IV route in cattle to ensure that PK/PD in this large animal system was comparable to the anticipated emodepside distribution in humans (*i*.*e*., *overall* exposure in blood including bound and unbound drug). However, we acknowledge that the rate of onset of systemic blood exposure would have been faster, and the C_max_ higher, in cattle compared with humans due to IV *versus* oral routes of administration, respectively. Finally, the maximum tolerable dose for Holstein cattle determined in Germany (emodepside 1 mg/kg with solketal as vehicle) was confirmed in the local breed of zebu (Ngaoundéré Gudali) cattle at the study site in Cameroon, although as a safety margin, only 75% of this dose was administered because of the increased environmental stresses on cattle in the tropics.

As was expected from prior *in vitro* and *in vivo* data on the effects of emodepside on *Onchocerca* spp. and other filariae [28–30,68], the drug exhibited dose-dependent efficacy against both Mf and adult worms. The kinetics of Mf clearance in the first few days after treatment at the higher dose were similar to those observed in humans or cattle treated with a single dose of IVM [59,69]. However, while some recovery of skin Mf densities was apparent, the 7-day regimens displayed a more prolonged suppressive effect on Mf than might have been expected with IVM [70], with an 87% reduction in pre-treatment Mf load in the EMO7H group by 12 months. Indeed, by the end of the experiment, there were indications that a permanent suppression of female worm fecundity may have been achieved in most of the emodepside-treated groups. Accordingly, although the embryogram results did not indicate a significant dose-dependent effect overall, it was clear that oocytes (a stage unaffected by IVM [4,71]) were eliminated in the multi-dose treatment groups.

The efficacy of emodepside against adult worm viability was more complex to interpret. In common with most laboratories conducting assays of macrofilaricidal activity, we applied a combination of motility scoring and quantification of MTT reduction to assess adult worm viability. While some laboratories have introduced image analysis algorithms to analyse motility [72–74], several other research groups continue to use subjective scoring systems as we applied here [19,75,76]. Increased automation is important for *in vitro* drug discovery projects to maximise throughput and reduce operator fatigue but is less frequently used for *ex vivo* analysis of worms from animal studies, as fewer parasites are usually involved at any one time. Importantly, automated motility assessment has never been evaluated on female *Onchocerca* spp. worms, which are very sluggish even in the absence of drug treatment, and *in vitro* drug screening with *O*. *ochengi* continues to use visual scoring, albeit with a more complex six-point categorisation than we applied here *ex vivo* [77]. The fact that subjective motility scoring by technicians blinded to treatment group indicated clear dose-dependent effects of emodepside against adult worms supports the reliability of this readout.

While all regimens had a significant negative impact on female worm motility, the effects of emodepside were very slow, and even in the EMO7H group, one animal harboured a female worm that still displayed some motility at the end of the experiment. Clearly, emodepside lacks the unambiguous and relatively rapid macrofilaricidal activity of melarsomine, which has been shown to kill most worms within three months [78]. Furthermore, several immotile female worms (including from animals in the EMO7H group) were still metabolically active in the MTT assay at the end of the experiment. Very slow killing of adult filariae is known to be highly advantageous, as drugs with comparatively rapid macrofilaricidal activity (killing within a few months), including melarsomine and flubendazole or their derivatives, are associated with intrinsic toxicity and can induce severe adverse events post treatment [79–81]. Conversely, the indirect mode of action of doxycycline against *O*. *volvulus* leads to a 70–80% reduction in worm lifespan (from ~10 years to 2–3 years) as well as permanent sterilisation of the females [82], and this ‘death by attrition’ is well tolerated by the host. Although it seems likely that the vast majority of female worms were sterilised and irreversibly damaged by the EMO7H regimen, we cannot rule out the possibility that the observed paralysis would eventually be overcome, perhaps with resumption of embryogenesis in some individuals. This potential disconnect between motility and viability (*i*.*e*., immotile worms are not necessarily dead) is not unexpected for a tissue-dwelling nematode, but the distinction is often ignored in evaluation of anthelmintics more generally because paralysed gastrointestinal nematodes are expelled in the faeces. Moreover, our observations over an 18-month experiment highlight the exceptionally chronic nature of the *Onchocerca* lifecycle and the resilience of the adult worms that can only be thoroughly assessed in natural hosts over an extended period.

Blood glucose has been identified as a simple biomarker for emodepside exposure in humans [83]. Hence, it was used successfully in this cattle study as a surrogate measure of emodepside exposure. In prior pharmacological analyses, the target of emodepside in mammals was not clear because both neurological symptoms and endocrine effects were observed in toxicological studies [84]. Furthermore, the effects on the nervous system were biphasic (excitatory then inhibitory). Proposed targeting of the GABAergic system remained inconclusive because emodepside showed no activity on GABA_A_ receptors or GABA transporters [26,84]. In addition, a reversible increase in blood glucose was observed in toxicological studies, which was triggered by a decrease in insulin and increase in glucagon excretion [83]. However, although catabolic dysfunctions comparable to a diabetic type I situation were reported in a rat model after prolonged, high-dose emodepside exposure (~4–5 mg/kg, over four weeks orally in diet, was defined as the no-observed-adverse-effect level [83]), it was not a dose-limiting effect in a recent first-in-human trial [54]. The minor impacts on blood glucose and insulin levels in humans were only observed under fasting conditions and were not considered as clinically significant or reported as an adverse event [83].

The actual target of emodepside is now known to be the human SLO-1 channel [39]—calcium-activated potassium channel subunit alpha-1 (KCNMA1). Emodepside’s action on KCNMA1 at 10–100 nM elicits a biphasic, concentration-dependent effect on the current amplitude, with transient facilitation followed by inhibition. These data align closely with the neurological findings; however, KCNMA1 is an important modulator of both neuronal activities [85] and secretion in the pancreatic ducts [86], explaining the link with glucose metabolism. Therefore, it is more likely that the modulatory effect on KCNMA1 is responsible for the side-effects of emodepside than direct effects on the GABA_A_ receptor. Note that in our assay where *Onchocerca* spp. SLO-1 channels were expressed in *Xenopus* oocytes, the minimum emodepside concentration required to induce a detectable increase in current (3 μM) was much higher than the concentrations that affect worm motility *in vitro* [30]. There are several potential reasons for this, including differences in drug solubility (only 0.1% DMSO was used in the *Xenopus* oocyte assay to prevent toxicity) and highly dissimilar assay timescales (milliseconds to minutes with oocytes; hours to weeks with worm assays). Certainly, there will also be marked differences in SLO-1 expression levels between these systems and the nature of the readouts was entirely distinct.

Several antifilarial drugs, including IVM, diethylcarbamazine and tetracycline antibiotics, have limited activity *in vitro*, requiring interactions with the immune system for optimal efficacy [87–90]; although a recent study demonstrated that diethylcarbamazine has direct effects on *B*. *malayi* by inducing temporary paralysis via transient receptor potential channels [74]. In the case of antibiotics, killing of adult *O*. *ochengi* by oxytetracycline (but not melarsomine) is correlated with eosinophil degranulation on the worm cuticle following depletion of *Wolbachia* [57,60]. However, emodepside in the nanomolar range has been shown to have macrofilaricidal activity *in vitro* against *Onchocerca* spp. [68] and we observed no clear relationship between emodepside dose and PMN infiltration into the nodule. Further studies are required to determine whether emodepside has any immunomodulatory activity and the extent to which the immune response may facilitate killing of Mf by this compound.

In conclusion, emodepside has embryotoxic and direct microfilaricidal activity against *O*. *ochengi in vivo*, with prolonged and possibly irreversible effects on adult female fecundity. The EMO7H regimen exhibited slow-acting and incomplete macrofilaricidal activity within the 18-month timeframe of this field trial, although the observed paralysis of the adult females may also be irreversible. Emodepside is now set to be evaluated in a phase II trial in Ghana in a range of regimens, including a single of dose of 30 mg, and daily or twice-daily doses of 15 mg for either 14 or 10 days, respectively [91]. Thus, our data clearly support emodepside’s current status as a priority macrofilaricidal candidate with the potential to accelerate the elimination of onchocerciasis without compromising patient safety.

## Supporting information

S1 Text Appendix containing Figures A-O and Tables A-I. **Fig A in S1 Text**. Overview of predicted *Onchocerca volvulus slo-1* splice variants. **Fig B in S1 Text**. Sequence alignment *Onchocerca volvulus* and *Onchocerca ochengi* SLO-1a splice variant (GenBank: MW039265). **Fig C in S1 Text**. Sequence alignment *Onchocerca volvulus* and *Onchocerca ochengi* SLO-1*b* splice variant (GenBank: MW039266). **Fig D in S1 Text**. PK analysis of emodepside concentration in cattle. **Fig E in S1 Text**. Ratio of skin to plasma concentrations of emodepside in zebu (Ngaoundéré Gudali) cattle (*B*. *t*. *indicus*) over time. **Fig F in S1 Text**. Dermal microfilarial density (*n* per 100 mg skin) in zebu (Ngaoundéré Gudali) cattle (*B*. *t*. *indicus*) treated with emodepside or melarsomine compared with placebo. **Fig G in S1 Text**. Mean numbers of (A) oocytes, (B) developing embryonic stages, (C) normal, and (D) degenerated intrauterine microfilariae in cattle treated with emodepside or melarsomine compared with placebo. **Fig H in S1 Text**. Effects of emodepside on adult female worm motility. **Fig I in S1 Text**. Effects of emodepside on adult female worm viability (MTT reduction). **Fig J in S1 Text**. Effects of emodepside on mean adult female worm fecundity in comparison with melarsomine and placebo. **Fig K in S1 Text**. Effects of emodepside on mean adult male worm motility in comparison with melarsomine and placebo. **Fig L in S1 Text**. Histopathology of *Onchocerca ochengi* nodules treated with emodepside. **Fig M in S1 Text**. Nodular polymorphonuclear counts per high-power field for each treatment group and timepoint. **Fig N in S1 Text**. Nodular eosinophil counts as a percentage of total polymorphonuclear cell counts for each treatment group and timepoint. **Fig O in S1 Text**. Linear model describing the relationship between change in adult male worm motility over time and total emodepside dose. **Table A in S1 Text**. Grading and scoring system for histopathological assessment of *Onchocerca ochengi* nodules [62]. **Table B in S1 Text**. Summary of PK parameters of non-compartmental analysis of the simulated concentration-time profiles after repeated administration of emodepside. **Table C in S1 Text**. Observed emodepside C_max_ and t_max_ in cattle. **Table D in S1 Text**. Summary statistics for histological sections examined by experimental group and timepoint. **Table E in S1 Text**. Mean histopathological scores (graded 1–4) for each specific anatomical component by treatment group and timepoint. **Table F in S1 Text**. Linear mixed-effects model results for cumulative histopathological worm scores analyzed (A) at each timepoint compared with the placebo control group and (B) within each treatment group compared with pre-treatment observations (day 1). **Table G in S1 Text**. Linear mixed-effects model results for nodular polymorphonuclear counts per high-power field analyzed (A) at each timepoint compared with the placebo control group and (B) within each treatment group compared with pre-treatment observations (day 1). **Table H in S1 Text**. Linear mixed-effects model results for nodular eosinophil counts as a percentage of total polymorphonuclear cell counts analyzed (A) at each timepoint compared with the placebo control group and (B) within each treatment group compared with pre-treatment observations (day 1). **Table I in S1 Text**. Fraction of normal adult worm motility (% [n]) by treatment group, sex, and timepoint.
(PDF)
