# Supplementary material for: Emodepside targets SLO-1 channels of Onchocerca ochengi and induces broad anthelmintic effects in a bovine model of onchocerciasis
Source: PLoS Pathog. 2021 Jun 2;17(6):e1009601. doi: 10.1371/journal.ppat.1009601 (PMC8202924; doi:10.1371/journal.ppat.1009601)
Supplement: S1 Text — Fig A in S1 Text. Overview of predicted Onchocerca volvulus slo-1 splice variants. Fig B in S1 Text. Sequence alignment Onchocerca volvulus and Onchocerca ochengi SLO-1a splice variant (GenBank: MW039265). Fig C in S1 Text. Sequence alignment Onchocerca volvulus and Onchocerca ochengi SLO-1b splice variant (GenBank: MW039266). Fig D in S1 Text. PK analysis of emodepside concentration in cattle. Fig E in S1 Text. Ratio of skin to plasma concentrations of emodepside in zebu (Ngaoundéré Gudali) cattle (B. t. indicus) over time. Fig F in S1 Text. Dermal microfilarial density (n per 100 mg skin) in zebu (Ngaoundéré Gudali) cattle (B. t. indicus) treated with emodepside or melarsomine compared with placebo. Fig G in S1 Text. Mean numbers of (A) oocytes, (B) developing embryonic stages, (C) normal, and (D) degenerated intrauterine microfilariae in cattle treated with emodepside or melarsomine compared with placebo. Fig H in S1 Text. Effects of emodepside on adult female worm motility. Fig I in S1 Text. Effects of emodepside on adult female worm viability (MTT reduction). Fig J in S1 Text. Effects of emodepside on mean adult female worm fecundity in comparison with melarsomine and placebo. Fig K in S1 Text. Effects of emodepside on mean adult male worm motility in comparison with melarsomine and placebo. Fig L in S1 Text. Histopathology of Onchocerca ochengi nodules treated with emodepside. Fig M in S1 Text. Nodular polymorphonuclear counts per high-power field for each treatment group and timepoint. Fig N in S1 Text. Nodular eosinophil counts as a percentage of total polymorphonuclear cell counts for each treatment group and timepoint. Fig O in S1 Text. Linear model describing the relationship between change in adult male worm motility over time and total emodepside dose. Table A in S1 Text. Grading and scoring system for histopathological assessment of Onchocerca ochengi nodules [62]. Table B in S1 Text. Summary of PK parameters of non-compartmental analysis of the simula [file ppat.1009601.s001.pdf]

Appendix

Figure A. Overview of predicted *Onchocerca volvulus slo-1* splice variants. 3

Figure B. Sequence alignment *Onchocerca volvulus* and *Onchocerca ochengi* SLO-1*a* splice variant (GenBank: MW039265). 4

Figure C. Sequence alignment *Onchocerca volvulus* and *Onchocerca ochengi* SLO-1*b* splice variant (GenBank: MW039266). 6

Figure D. PK analysis of emodepside concentration in cattle. 8

Figure E. Ratio of skin to plasma concentrations of emodepside in zebu (Ngaoundéré Gudali) cattle (*B. t. indicus*) over time. 10

Figure F. Dermal microfilarial density (*n* per 100 mg skin) in zebu (Ngaoundéré Gudali) cattle (*B. t. indicus*) treated with emodepside or melarsomine compared with placebo. 11

Figure G. Mean numbers of (a) oocytes, (b) developing embryonic stages, (c) normal, and (d) degenerated intrauterine microfilariae in cattle treated with emodepside or melarsomine compared with placebo. 13

Figure H. Effects of emodepside on adult female worm motility. 15

Figure I. Effects of emodepside on adult female worm viability (MTT reduction). 17

Figure J. Effects of emodepside on mean adult female worm fecundity in comparison with melarsomine and placebo. 19

Figure K. Effects of emodepside on mean adult male worm motility in comparison with melarsomine and placebo. 20

Figure L. Histopathology of *Onchocerca ochengi* nodules treated with emodepside. 21

Figure M. Nodular polymorphonuclear counts per high-power field for each treatment group and timepoint. 22

Figure N. Nodular eosinophil counts as a percentage of total polymorphonuclear cell counts for each treatment group and timepoint. 23

Figure O. Linear model describing the relationship between change in adult male worm motility over time and total emodepside dose. 24

Table A. Grading and scoring system for histopathological assessment of *Onchocerca ochengi* nodules. 25

Table B. Summary of PK parameters of non-compartmental analysis of the simulated concentration-time profiles after repeated administration of emodepside. 26

Table C. Observed emodepside C_{max} and T_{max} in cattle. 27

Table D. Summary statistics for histological sections examined by experimental group and timepoint. 28

Table E. Mean histopathological scores (graded 1–4) for each specific anatomical component by treatment group and timepoint. 29

Table F. Linear mixed-effects model results for cumulative histopathological worm scores analysed (a) at each timepoint compared with the placebo control group and (b) within each treatment group compared with pre-treatment observations (day 1). 30

Table G. Linear mixed-effects model results for nodular polymorphonuclear counts per high-power field analysed (a) at each timepoint compared with the placebo control group and (b) within each treatment group compared with pre-treatment observations (day 1). 31

Table H. Linear mixed-effects model results for nodular eosinophil counts as a percentage of total polymorphonuclear cell counts analysed (a) at each timepoint compared with the placebo control group and (b) within each treatment group compared with pre-treatment observations (day 1)... 32

Table I. Fraction of normal adult worm motility (% [*n*]) by treatment group, sex, and timepoint. 33

Reference 34

Figure A. Overview of predicted *Onchocerca volvulus slo-1* splice variants.

The *O. volvulus slo-1* gene has five predicted full-length splice variants (WormBase CDS OVO4127*a*, *b*, *c*, *d*, *f*) and one truncated splice variant (WormBase CDS OVO4127*e*). In the diagram, exons are shown as blue boxes, intron regions as black lines. The full-length splice variants differ in the presence or absence of exons 14, 15, and 19 (dotted boxes). The *Ovo*_slo-1*f* splice variant has the longest coding sequence (1,143 amino acids), followed by splice variants *a* and *b* (1,119 amino acids each). Splice variants *c* and *d* both consist of 1,104 amino acids. The truncated splice variant *e* (375 amino acids) lacks exons 1 to 20.

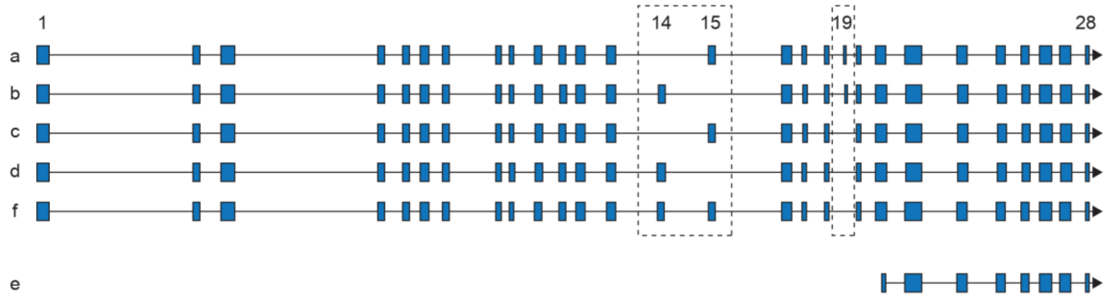

Figure B. Sequence alignment *Onchocerca volvulus* and *Onchocerca ochengi* SLO-1*a* splice variant (GenBank: MW039265).

Sequence alignments revealed that one of the two identified *O. ochengi* SLO-1 sequences has the highest similarity to the *O. volvulus* SLO-1 splice variant *a*.

Both splice variants have a length of 1,119 amino acids and differ only at position 658 (Arg [R] →Lys [K]; shown in red text).

```
                   10         20         30         40         50         60         70         80         90        100
Ooc_SLO-1a MSDVYHPGSG GSKGFPPAPY GYPMHCNMSR AFQEMTDDER KCLDERKYWC FLLSSIVTFC VSMLLVVIWR IIAHVFCQQR ERDEIDTVFD QDEGKHELFK
Ovo_SLO-1a MSDVYHPGSG GSKGFPPAPY GYPMHCNMSR AFQEMTDDER KCLDERKYWC FLLSSIVTFC VSMLLVVIWR IIAHVFCQQR ERDEIDTVFD QDEGKHELFK
Consensus  MSDVYHPGSG GSKGFPPAPY GYPMHCNMSR AFQEMTDDER KCLDERKYWC FLLSSIVTFC VSMLLVVIWR IIAHVFCQQR ERDEIDTVFD QDEGKHELFK

                  110        120        130        140        150        160        170        180        190        200
Ooc_SLO-1a GDGDGITIKQ EEVQIGWMTE AKDWAGELIS GQSMTGRILV VLVFLLSIGS LIIYFYDASH PNFQVETCIA WSDSPSQQID LGFNIFFLIY FFIRFIAASD
Ovo_SLO-1a GDGDGITIKQ EEVQIGWMTE AKDWAGELIS GQSMTGRILV VLVFLLSIGS LIIYFYDASH PNFQVETCIA WSDSPSQQID LGFNIFFLIY FFIRFIAASD
Consensus  GDGDGITIKQ EEVQIGWMTE AKDWAGELIS GQSMTGRILV VLVFLLSIGS LIIYFYDASH PNFQVETCIA WSDSPSQQID LGFNIFFLIY FFIRFIAASD

                  210        220        230        240        250        260        270        280        290        300
Ooc_SLO-1a KVWFLLEVYS FIDYFTIPPS FVAIYLERNW LGLRFLRALR LMTVPDILQY LNVLKTSSSI RLTQLLSIFI SVCLTGAGFV HVLENSGDPF KNFANTHRIT
Ovo_SLO-1a KVWFLLEVYS FIDYFTIPPS FVAIYLERNW LGLRFLRALR LMTVPDILQY LNVLKTSSSI RLTQLLSIFI SVCLTGAGFV HVLENSGDPF KNFANTHRIT
Consensus  KVWFLLEVYS FIDYFTIPPS FVAIYLERNW LGLRFLRALR LMTVPDILQY LNVLKTSSSI RLTQLLSIFI SVCLTGAGFV HVLENSGDPF KNFANTHRIT

                  310        320        330        340        350        360        370        380        390        400
Ooc_SLO-1a YWDCVYFLLV TMSTVGYGDI YCTTFLGRLF MVFFILGGLA MFASYIPEIA DLIGSRQKYG GEYKGEHGKK HIVVCGYITY ESVSHFLQDF LHEDREDVDV
Ovo_SLO-1a YWDCVYFLLV TMSTVGYGDI YCTTFLGRLF MVFFILGGLA MFASYIPEIA DLIGSRQKYG GEYKGEHGKK HIVVCGYITY ESVSHFLQDF LHEDREDVDV
Consensus  YWDCVYFLLV TMSTVGYGDI YCTTFLGRLF MVFFILGGLA MFASYIPEIA DLIGSRQKYG GEYKGEHGKK HIVVCGYITY ESVSHFLQDF LHEDREDVDV

                  410        420        430        440        450        460        470        480        490        500
Ooc_SLO-1a EVVFLHRVPP DLELEGLFKR HFTKVEFFSG TVMDSIDLSR VKVDEADACL VLANKYSSDP DAEDAANIMR VISIKNYSSD IRVIVQLMQY HNKAYLLNIP
Ovo_SLO-1a EVVFLHRVPP DLELEGLFKR HFTKVEFFSG TVMDSIDLSR VKVDEADACL VLANKYSSDP DAEDAANIMR VISIKNYSSD IRVIVQLMQY HNKAYLLNIP
Consensus  EVVFLHRVPP DLELEGLFKR HFTKVEFFSG TVMDSIDLSR VKVDEADACL VLANKYSSDP DAEDAANIMR VISIKNYSSD IRVIVQLMQY HNKAYLLNIP

                  510        520        530        540        550        560        570        580        590        600
Ooc_SLO-1a SWDWRRGDDV ICLAELKLGF IAQSCLAPGF STMMANLFAM RSFKTSRNTP DWLNLYLCGA GMEMYTDTLS HGFVGMTFPE AADLLFTRLG LLLLAIELKD
Ovo_SLO-1a SWDWRRGDDV ICLAELKLGF IAQSCLAPGF STMMANLFAM RSFKTSRNTP DWLNLYLCGA GMEMYTDTLS HGFVGMTFPE AADLLFTRLG LLLLAIELKD
Consensus  SWDWRRGDDV ICLAELKLGF IAQSCLAPGF STMMANLFAM RSFKTSRNTP DWLNLYLCGA GMEMYTDTLS HGFVGMTFPE AADLLFTRLG LLLLAIELKD

                  610        620        630        640        650        660        670        680        690        700
Ooc_SLO-1a DEKKECNIAI NPAPTTTIQP QTQGFFIAQS ADEVKRAFYW CKQCHEDIID VSLIKKCKCR NLNLFRKGVK QVQLSKRIDG DPKNSYLNAL DDIARATPVS
Ovo_SLO-1a DEKKECNIAI NPAPTTTIQP QTQGFFIAQS ADEVKRAFYW CKQCHEDIID VSLIKKCRCR NLNLFRKGVK QVQLSKRIDG DPKNSYLNAL DDIARATPVS
Consensus  DEKKECNIAI NPAPTTTIQP QTQGFFIAQS ADEVKRAFYW CKQCHEDIID VSLIKKCrCR NLNLFRKGVK QVQLSKRIDG DPKNSYLNAL DDIARATPVS
```

```
                    710        720        730        740        750        760        770        780        790        800
Ooc_SLO-1a NASNDTTVQL RRLDDRRSYG RKNSLSLPPE GRTIDFTKDF EQQDMKYDST GMFHWCPART LEECVLDRNQ AAMTVLNGHV VVCLFADRNS PLIGLRNFVM
Ovo_SLO-1a NASNDTTVQL RRLDDRRSYG RKNSLSLPPE GRTIDFTKDF EQQDMKYDST GMFHWCPART LEECVLDRNQ AAMTVLNGHV VVCLFADRNS PLIGLRNFVM
Consensus  NASNDTTVQL RRLDDRRSYG RKNSLSLPPE GRTIDFTKDF EQQDMKYDST GMFHWCPART LEECVLDRNQ AAMTVLNGHV VVCLFADRNS PLIGLRNFVM

                    810        820        830        840        850        860        870        880        890        900
Ooc_SLO-1a PLRASNFHYH ELKHVVIVGD VEYLKREWKT LFNLPKISIL NGSPLSRADL RAVNINLCDM CVIVSARIPN PNEDPTLADK EAILASLNIK AMQFDDTLGF
Ovo_SLO-1a PLRASNFHYH ELKHVVIVGD VEYLKREWKT LFNLPKISIL NGSPLSRADL RAVNINLCDM CVIVSARIPN PNEDPTLADK EAILASLNIK AMQFDDTLGF
Consensus  PLRASNFHYH ELKHVVIVGD VEYLKREWKT LFNLPKISIL NGSPLSRADL RAVNINLCDM CVIVSARIPN PNEDPTLADK EAILASLNIK AMQFDDTLGF

                    910        920        930        940        950        960        970        980        990       1000
Ooc_SLO-1a YPLRQTGGAV SPLGSPLSLQ KKGARFGTNV PMITELVNDS NVQFLDQDDD DDPDTELYLT QPFACGTAFA ISVLDSLMST TYFNDSALTL IRTLVTGGAT
Ovo_SLO-1a YPLRQTGGAV SPLGSPLSLQ KKGARFGTNV PMITELVNDS NVQFLDQDDD DDPDTELYLT QPFACGTAFA ISVLDSLMST TYFNDSALTL IRTLVTGGAT
Consensus  YPLRQTGGAV SPLGSPLSLQ KKGARFGTNV PMITELVNDS NVQFLDQDDD DDPDTELYLT QPFACGTAFA ISVLDSLMST TYFNDSALTL IRTLVTGGAT

                   1010       1020       1030       1040       1050       1060       1070       1080       1090       1100
Ooc_SLO-1a PEMELILAEG AGLRGGYSTP ETLNNRDRCR ISQLALQDRP FEGITTGSSY GQMFSIALKR HGQLCIGLYR LHDQAAVDSN KRYVITNPPA ELRLLLSDYV
Ovo_SLO-1a PEMELILAEG AGLRGGYSTP ETLNNRDRCR ISQLALQDRP FEGITTGSSY GQMFSIALKR HGQLCIGLYR LHDQAAVDSN KRYVITNPPA ELRLLLSDYV
Consensus  PEMELILAEG AGLRGGYSTP ETLNNRDRCR ISQLALQDRP FEGITTGSSY GQMFSIALKR HGQLCIGLYR LHDQAAVDSN KRYVITNPPA ELRLLLSDYV

                   1110
Ooc_SLO-1a YVLEQFDPGL EYEPRKNFL
Ovo_SLO-1a YVLEQFDPGL EYEPRKNFL
Consensus  YVLEQFDPGL EYEPRKNFL
```

Figure C. Sequence alignment *Onchocerca volvulus* and *Onchocerca ochengi* SLO-1*b* splice variant (GenBank: MW039266).

Sequence alignments revealed that one of the two identified *O. ochengi* SLO-1 sequences has the highest similarity to the *O. volvulus* SLO-1 splice variant *b*. Both splice variants have a length of 1,119 amino acids and differ only at positions 658 (Arg [R]→Lys [K]) and 711 (Arg [R]→Cys [C]). Both amino acid substitutions are shown in red text.

```
                    10         20         30         40         50         60         70         80         90        100
Ooc_SLO-1b MSDVYHPGSG GSKGFPPAPY GYPMHCNMSR AFQEMTDDER KCLDERKYWC FLLSSIVTFC VSMLLVVIWR IIAHVFCQQR ERDEIDTVFD QDEGKHELFK
Ovo_SLO-1b MSDVYHPGSG GSKGFPPAPY GYPMHCNMSR AFQEMTDDER KCLDERKYWC FLLSSIVTFC VSMLLVVIWR IIAHVFCQQR ERDEIDTVFD QDEGKHELFK
Consensus  MSDVYHPGSG GSKGFPPAPY GYPMHCNMSR AFQEMTDDER KCLDERKYWC FLLSSIVTFC VSMLLVVIWR IIAHVFCQQR ERDEIDTVFD QDEGKHELFK

                   110        120        130        140        150        160        170        180        190        200
Ooc_SLO-1b GDGDGITIKQ EEVQIGWMTE AKDWAGELIS GQSMTGRILV VLVFLLSIGS LIIYFYDASH PNFQVETCIA WSDSPSQQID LGFNIFFLIY FFIRFIAASD
Ovo_SLO-1b GDGDGITIKQ EEVQIGWMTE AKDWAGELIS GQSMTGRILV VLVFLLSIGS LIIYFYDASH PNFQVETCIA WSDSPSQQID LGFNIFFLIY FFIRFIAASD
Consensus  GDGDGITIKQ EEVQIGWMTE AKDWAGELIS GQSMTGRILV VLVFLLSIGS LIIYFYDASH PNFQVETCIA WSDSPSQQID LGFNIFFLIY FFIRFIAASD

                   210        220        230        240        250        260        270        280        290        300
Ooc_SLO-1b KVWFLLEVYS FIDYFTIPPS FVAIYLERNW LGLRFLRALR LMTVPDILQY LNVLKTSSSI RLTQLLSIFI SVCLTGAGFV HVLENSGDPF KNFANTHRIT
Ovo_SLO-1b KVWFLLEVYS FIDYFTIPPS FVAIYLERNW LGLRFLRALR LMTVPDILQY LNVLKTSSSI RLTQLLSIFI SVCLTGAGFV HVLENSGDPF KNFANTHRIT
Consensus  KVWFLLEVYS FIDYFTIPPS FVAIYLERNW LGLRFLRALR LMTVPDILQY LNVLKTSSSI RLTQLLSIFI SVCLTGAGFV HVLENSGDPF KNFANTHRIT

                   310        320        330        340        350        360        370        380        390        400
Ooc_SLO-1b YWDCVYFLLV TMSTVGYGDI YCTTFLGRLF MVFFILGGLA MFASYIPEIA DLIGSRQKYG GEYKGEHGKK HIVVCGYITY ESVSHFLQDF LHEDREDVDV
Ovo_SLO-1b YWDCVYFLLV TMSTVGYGDI YCTTFLGRLF MVFFILGGLA MFASYIPEIA DLIGSRQKYG GEYKGEHGKK HIVVCGYITY ESVSHFLQDF LHEDREDVDV
Consensus  YWDCVYFLLV TMSTVGYGDI YCTTFLGRLF MVFFILGGLA MFASYIPEIA DLIGSRQKYG GEYKGEHGKK HIVVCGYITY ESVSHFLQDF LHEDREDVDV

                   410        420        430        440        450        460        470        480        490        500
Ooc_SLO-1b EVVFLHRVPP DLELEGLFKR HFTKVEFFSG TVMDSIDLSR VKVDEADACL VLANKYSSDP DAEDAANIMR VISIKNYSSD IRVIVQLMQY HNKAYLLNIP
Ovo_SLO-1b EVVFLHRVPP DLELEGLFKR HFTKVEFFSG TVMDSIDLSR VKVDEADACL VLANKYSSDP DAEDAANIMR VISIKNYSSD IRVIVQLMQY HNKAYLLNIP
Consensus  EVVFLHRVPP DLELEGLFKR HFTKVEFFSG TVMDSIDLSR VKVDEADACL VLANKYSSDP DAEDAANIMR VISIKNYSSD IRVIVQLMQY HNKAYLLNIP

                   510        520        530        540        550        560        570        580        590        600
Ooc_SLO-1b SWDWRRGDDV ICLAELKLGF IAQSCLAPGF STMMANLFAM RSFKTSPHTP QWLNDYLRGA GMEMYTESLS HSCVGMTFTE AADLLFTRLG LLLLAIELKD
Ovo_SLO-1b SWDWRRGDDV ICLAELKLGF IAQSCLAPGF STMMANLFAM RSFKTSPHTP QWLNDYLRGA GMEMYTESLS HSCVGMTFTE AADLLFTRLG LLLLAIELKD
Consensus  SWDWRRGDDV ICLAELKLGF IAQSCLAPGF STMMANLFAM RSFKTSPHTP QWLNDYLRGA GMEMYTESLS HSCVGMTFTE AADLLFTRLG LLLLAIELKD

                   610        620        630        640        650        660        670        680        690        700
Ooc_SLO-1b DEKKECNIAI NPAPTTTIQP QTQGFFIAQS ADEVKRAFYW CKQCHEDIID VSLIKKCKCR NLNLFRKGVK QVQLSKRIDG DPKNSYLNAL DDIARATPVS
Ovo_SLO-1b DEKKECNIAI NPAPTTTIQP QTQGFFIAQS ADEVKRAFYW CKQCHEDIID VSLIKKCRCR NLNLFRKGVK QVQLSKRIDG DPKNSYLNAL DDIARATPVS
Consensus  DEKKECNIAI NPAPTTTIQP QTQGFFIAQS ADEVKRAFYW CKQCHEDIID VSLIKKCrCR NLNLFRKGVK QVQLSKRIDG DPKNSYLNAL DDIARATPVS
```

```
                    710        720        730        740        750        760        770        780        790        800
Ooc_SLO-1b NASNDTTVQL CRLDDRRSYG RKNSLSLPPE GRTIDFTKDF EQQDMKYDST GMFHWCPART LEECVLDRNQ AAMTVLNGHV VVCLFADRNS PLIGLRNFVM
Ovo_SLO-1b NASNDTTVQL RRLDDRRSYG RKNSLSLPPE GRTIDFTKDF EQQDMKYDST GMFHWCPART LEECVLDRNQ AAMTVLNGHV VVCLFADRNS PLIGLRNFVM
Consensus  NASNDTTVQL rRLDDRRSYG RKNSLSLPPE GRTIDFTKDF EQQDMKYDST GMFHWCPART LEECVLDRNQ AAMTVLNGHV VVCLFADRNS PLIGLRNFVM

                    810        820        830        840        850        860        870        880        890        900
Ooc_SLO-1b PLRASNFHYH ELKHVVIVGD VEYLKREWKT LFNLPKISIL NGSPLSRADL RAVNINLCDM CVIVSARIPN PNEDPTLADK EAILASLNIK AMQFDDTLGF
Ovo_SLO-1b PLRASNFHYH ELKHVVIVGD VEYLKREWKT LFNLPKISIL NGSPLSRADL RAVNINLCDM CVIVSARIPN PNEDPTLADK EAILASLNIK AMQFDDTLGF
Consensus  PLRASNFHYH ELKHVVIVGD VEYLKREWKT LFNLPKISIL NGSPLSRADL RAVNINLCDM CVIVSARIPN PNEDPTLADK EAILASLNIK AMQFDDTLGF

                    910        920        930        940        950        960        970        980        990       1000
Ooc_SLO-1b YPLRQTGGAV SPLGSPLSLQ KKGARFGTNV PMITELVNDS NVQFLDQDDD DDPDTELYLT QPFACGTAFA ISVLDSLMST TYFNDSALTL IRTLVTGGAT
Ovo_SLO-1b YPLRQTGGAV SPLGSPLSLQ KKGARFGTNV PMITELVNDS NVQFLDQDDD DDPDTELYLT QPFACGTAFA ISVLDSLMST TYFNDSALTL IRTLVTGGAT
Consensus  YPLRQTGGAV SPLGSPLSLQ KKGARFGTNV PMITELVNDS NVQFLDQDDD DDPDTELYLT QPFACGTAFA ISVLDSLMST TYFNDSALTL IRTLVTGGAT

                   1010       1020       1030       1040       1050       1060       1070       1080       1090       1100
Ooc_SLO-1b PEMELILAEG AGLRGGYSTP ETLNNRDRCR ISQLALQDRP FEGITTGSSY GQMFSIALKR HGQLCIGLYR LHDQAAVDSN KRYVITNPPA ELRLLLSDYV
Ovo_SLO-1b PEMELILAEG AGLRGGYSTP ETLNNRDRCR ISQLALQDRP FEGITTGSSY GQMFSIALKR HGQLCIGLYR LHDQAAVDSN KRYVITNPPA ELRLLLSDYV
Consensus  PEMELILAEG AGLRGGYSTP ETLNNRDRCR ISQLALQDRP FEGITTGSSY GQMFSIALKR HGQLCIGLYR LHDQAAVDSN KRYVITNPPA ELRLLLSDYV

                   1110
Ooc_SLO-1b YVLEQFDPGL EYEPRKNFL
Ovo_SLO-1b YVLEQFDPGL EYEPRKNFL
Consensus  YVLEQFDPGL EYEPRKNFL
```

Figure D. PK analysis of emodepside concentration in cattle.

(a) Simulated plasma concentration–time profiles of emodepside in cattle over one week after repeated daily administration of 0.15 mg/kg (“humanized” dose) or 0.75 mg/kg via 15 min IV infusion based on a PK studies in two Holstein cows (receiving 1 mg/kg and 2.13 mg/kg respectively).

(b) Observed plasma concentration of emodepside in zebu cattle (datapoints) receiving once-daily IV emodepside 0.15 mg/kg, 0.15 mg/kg for 2 days, 0.75 mg/kg, or placebo (data not shown), and predicted concentration–time profiles after model fitting (red lines) of data from PK studies in two Holstein cows. Emodepside concentration in plasma was determined by tandem mass spectrometry; lower limit of quantitation was 1.0 μg/L. All samples in the placebo control were below the lower limit of quantitation. No statistical comparisons were made.

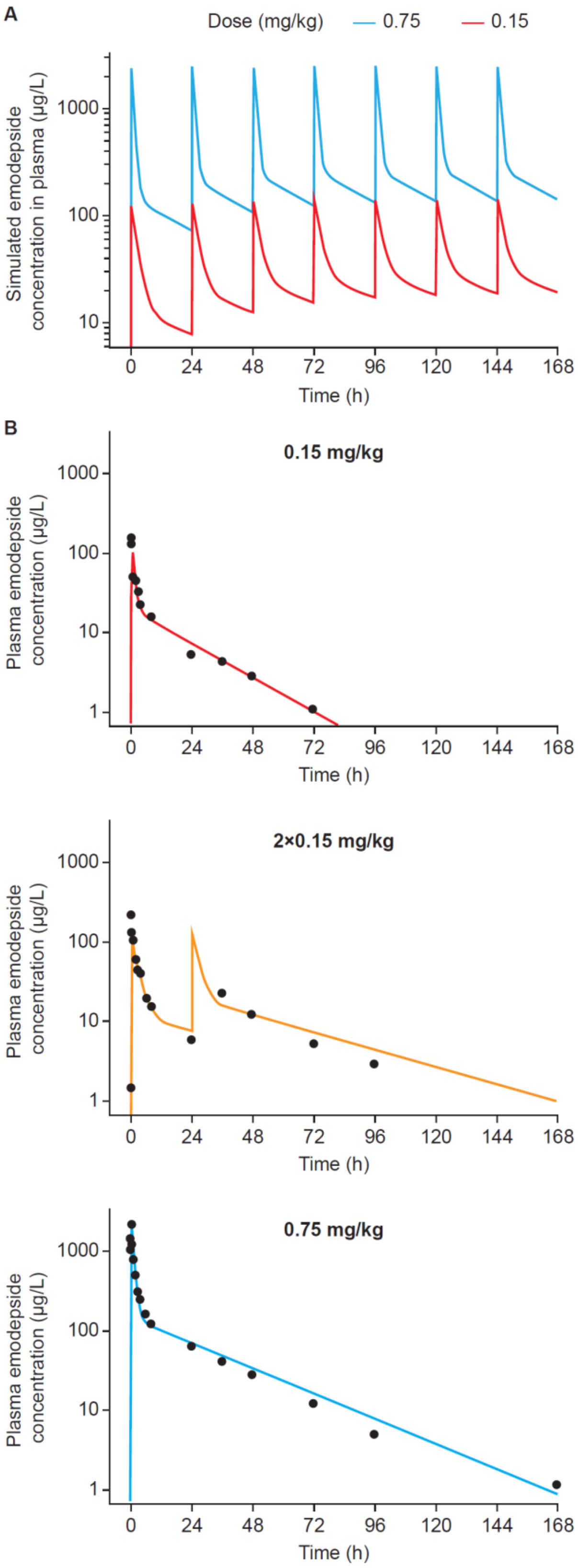
A
Dose (mg/kg)
0.75
0.15
Simulated emodepside concentration in plasma (µg/L)
1000
100
10
0
24
48
72
96
120
144
168
Time (h)
B
0.15 mg/kg
Plasma emodepside concentration (µg/L)
1000
100
10
1
Time (h)
2×0.15 mg/kg
0.75 mg/kg

Figure E. Ratio of skin to plasma concentrations of emodepside in zebu (Ngaoundéré Gudali) cattle (*B. t. indicus*) over time.

Observations in the same animal are connected by colored lines. The black horizontal line indicates a ratio of 1; the overall median ratio was 1.1. The *y*-axis is a logarithmic scale. No statistical comparisons were made.

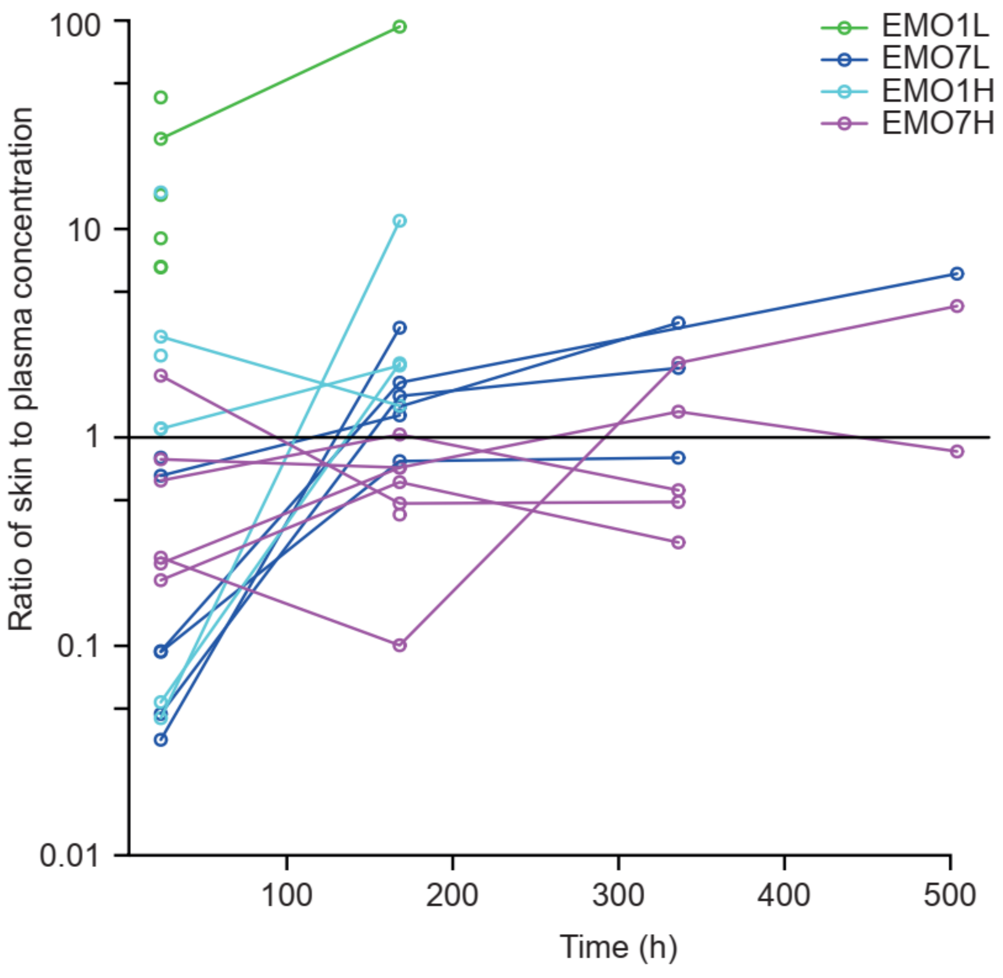

EMO1H, emodepside, 0.75 mg/kg, single dose; EMO7H, emodepside, 0.75 mg/kg, daily for seven days; EMO1L, emodepside, 0.15 mg/kg, single dose; EMO7L, emodepside, 0.15 mg/kg, daily for seven days.

Figure F. Dermal microfilarial density (*n* per 100 mg skin) in zebu (Ngaoundéré Gudali) cattle (*B. t. indicus*) treated with emodepside or melarsomine compared with placebo.

Dermal microfilarial density was determined by incubation of skin biopsies (in triplicate per animal, per timepoint) and normalised to 100 mg skin [1]. Density values are shown at each timepoint in each treatment group and are color-coded by quantile, from pale yellow (low microfilarial density) to dark red (high microfilarial density). The data are sorted in six equally sized bins; owing to its frequency, the value 0 occupies more than one bin, therefore the shading of cells with value 0 can be one of two colours. No between-group statistical comparisons were made.

EMO1H, emodepside, 0.75 mg/kg, single dose; EMO7H, emodepside, 0.75 mg/kg, daily for seven days; EMO1L, emodepside, 0.15 mg/kg, single dose; EMO7L, emodepside, 0.15 mg/kg, daily for seven days; MRSM, melarsomine, 4 mg/kg, every other day for three days; NR, not recorded; PCBO, placebo.

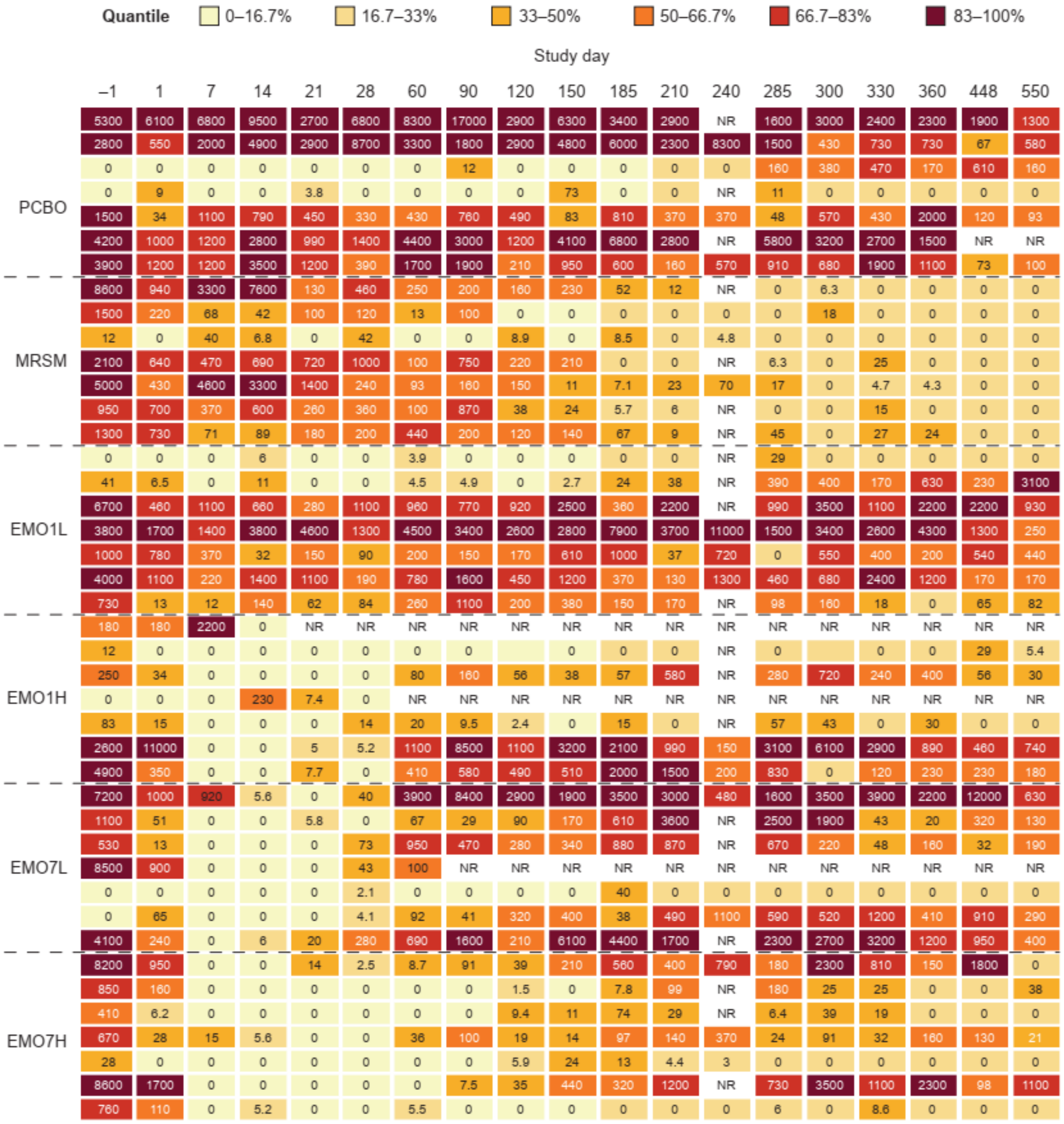

	–1	1	7	14	21	28	60	90	120	150	185	210	240	285	300	330	360	448	550
	5300	6100	6800	9500	2700	6800	8300	17000	2900	6300	3400	2900	NR	1600	3000	2400	2300	1900	1300
	2800	550	2000	4900	2900	8700	3300	1800	2900	4800	6000	2300	8300	1500	430	730	730	67	580
	0	0	0	0	0	0	0	12	0	0	0	0	0	160	380	470	170	610	160
PCBO	0	9	0	0	3.8	0	0	0	0	73	0	0	NR	11	0	0	0	0	0
	1500	34	1100	790	450	330	430	760	490	83	810	370	370	48	570	430	2000	120	93
	4200	1000	1200	2800	990	1400	4400	3000	1200	4100	6800	2800	NR	5800	3200	2700	1500	NR	NR
	3900	1200	1200	3500	1200	390	1700	1900	210	950	600	160	570	910	680	1900	1100	73	100
	8600	940	3300	7600	130	460	250	200	160	230	52	12	NR	0	6.3	0	0	0	0
	1500	220	68	42	100	120	13	100	0	0	0	0	0	0	18	0	0	0	0
	12	0	40	6.8	0	42	0	0	8.9	0	8.5	0	4.8	0	0	0	0	0	0
MRSM	2100	640	470	690	720	1000	100	750	220	210	0	0	NR	6.3	0	25	0	0	0
	5000	430	4600	3300	1400	240	93	160	150	11	7.1	23	70	17	0	4.7	4.3	0	0
	950	700	370	600	260	360	100	870	38	24	5.7	6	NR	0	0	15	0	0	0
	1300	730	71	89	180	200	440	200	120	140	67	9	NR	45	0	27	24	0	0
	0	0	0	6	0	0	3.9	0	0	0	0	0	NR	29	0	0	0	0	0
	41	6.5	0	11	0	0	4.5	4.9	0	2.7	24	38	NR	390	400	170	630	230	3100
	6700	460	1100	660	280	1100	960	770	920	2500	360	2200	NR	990	3500	1100	2200	2200	930
EMO1L	3800	1700	1400	3800	4600	1300	4500	3400	2600	2800	7900	3700	11000	1500	3400	2600	4300	1300	250
	1000	780	370	32	150	90	200	150	170	610	1000	37	720	0	550	400	200	540	440
	4000	1100	220	1400	1100	190	780	1600	450	1200	370	130	1300	460	680	2400	1200	170	170
	730	13	12	140	62	84	260	1100	200	380	150	170	NR	98	160	18	0	65	82
	180	180	2200	0	NR	NR	NR	NR	NR	NR	NR	NR	NR	NR	NR	NR	NR	NR	NR
	12	0	0	0	0	0	0	0		0	0	0	NR	0		0	0	29	5.4
	250	34	0	0	0	0	80	160	56	38	57	580	NR	280	720	240	400	56	30
EMO1H	0	0	0	230	7.4	0	NR	NR	NR	NR	NR	NR	NR	NR	NR	NR	NR	NR	NR
	83	15	0	0	0	14	20	9.5	2.4	0	15	0	NR	57	43	0	30	0	0
	2600	11000	0	0	5	5.2	1100	8500	1100	3200	2100	990	150	3100	6100	2900	890	460	740
	4900	350	0	0	7.7	0	410	580	490	510	2000	1500	200	830	0	120	230	230	180
	7200	1000	920	5.6	0	40	3900	8400	2900	1900	3500	3000	480	1600	3500	3900	2200	12000	630
	1100	51	0	0	5.8	0	67	29	90	170	610	3600	NR	2500	1900	43	20	320	130
	530	13	0	0	0	73	950	470	280	340	880	870	NR	670	220	48	160	32	190
EMO7L	8500	900	0	0	0	43	100	NR	NR	NR	NR	NR	NR	NR	NR	NR	NR	NR	NR
	0	0	0	0	0	2.1	0	0	0	0	40	0	0	0	0	0	0	0	0
	0	65	0	0	0	4.1	92	41	320	400	38	490	1100	590	520	1200	410	910	290
	4100	240	0	6	20	280	690	1600	210	6100	4400	1700	NR	2300	2700	3200	1200	950	400
	8200	950	0	0	14	2.5	8.7	91	39	210	560	400	790	180	2300	810	150	1800	0
	850	160	0	0	0	0	0	0	1.5	0	7.8	99	NR	180	25	25	0	0	38
	410	6.2	0	0	0	0	0	0	9.4	11	74	29	NR	6.4	39	19	0	0	0
EMO7H	670	28	15	5.6	0	0	36	100	19	14	97	140	370	24	91	32	160	130	21
	28	0	0	0	0	0	0	0	5.9	24	13	4.4	3	0	0	0	0	0	0
	8600	1700	0	0	0	0	0	7.5	35	440	320	1200	NR	730	3500	1100	2300	98	1100
	760	110	0	5.2	0	0	5.5	0	0	0	0	0	0	6	0	8.6	0	0	0

Figure G. Mean numbers of (a) oocytes, (b) developing embryonic stages, (c) normal, and (d) degenerated intrauterine microfilariae in cattle treated with emodepside or melarsomine compared with placebo.

Uterine counts (including intrauterine microfilariae) were made after homogenizing the posterior part of the female worm and counting the stages in a standardised manner in a Fuchs-Rosenthal chamber. Values are shown at each timepoint in each treatment group and are color-coded by quantile, from pale yellow (low) to dark red (high). Dendrograms were created by hierarchical clustering based on Euclidean distance between values over time and indicate the degree of similarity between treatments based on the *in vivo* response over time. No between-group statistical comparisons were made.

EMO1H, emodepside, 0.75 mg/kg, single dose; EMO7H, emodepside, 0.75 mg/kg, daily for seven days; EMO1L, emodepside, 0.15 mg/kg, single dose; EMO7L, emodepside, 0.15 mg/kg, daily for seven days; MRSM, melarsomine, 4 mg/kg, every other day for three days; PCBO, placebo.

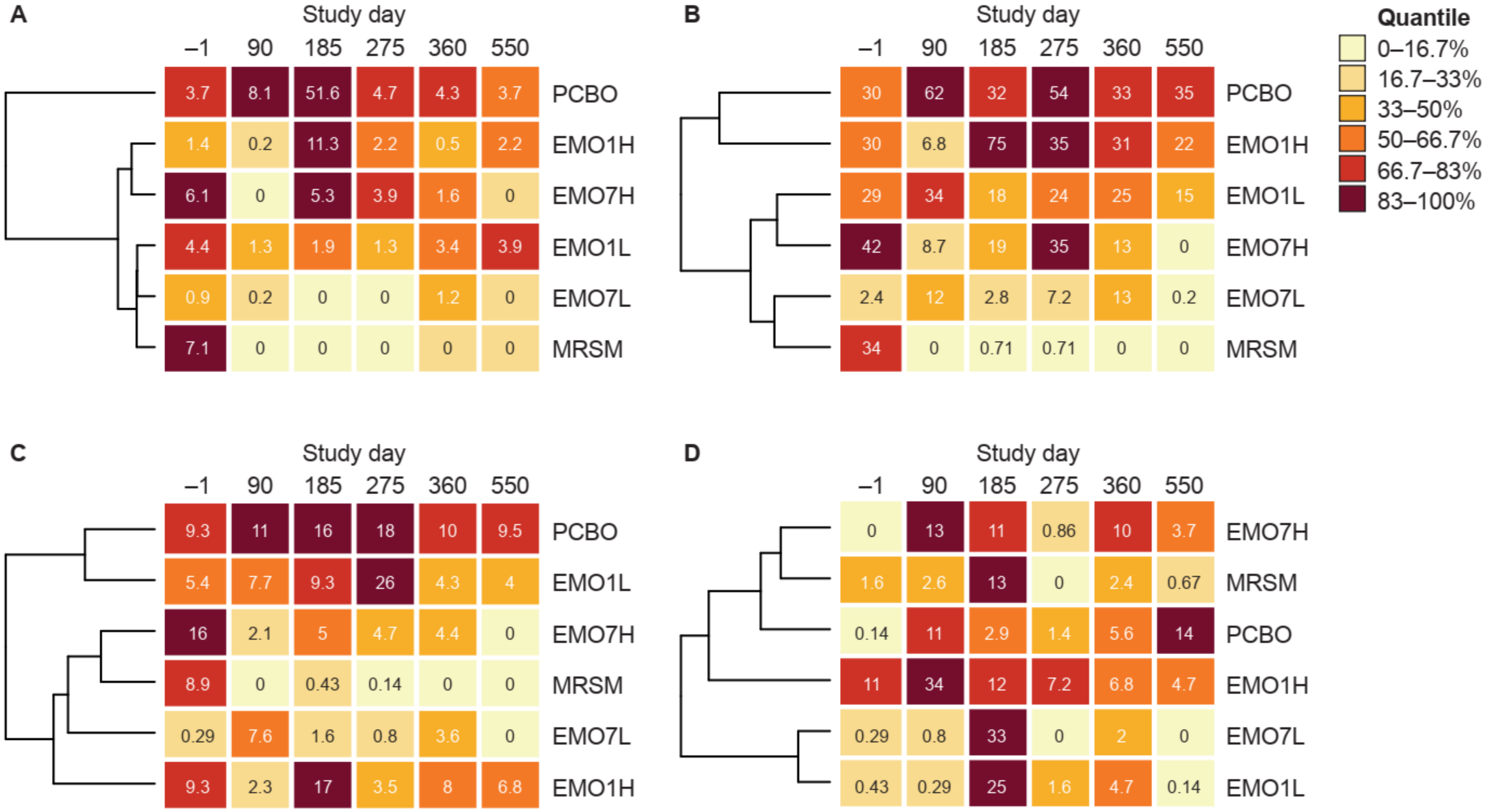
A
Study day
–1 90 185 275 360 550
3.7 8.1 51.6 4.7 4.3 3.7 PCBO
1.4 0.2 11.3 2.2 0.5 2.2 EMO1H
6.1 0 5.3 3.9 1.6 0 EMO7H
4.4 1.3 1.9 1.3 3.4 3.9 EMO1L
0.9 0.2 0 0 1.2 0 EMO7L
7.1 0 0 0 0 0 MRSM
B
Study day
–1 90 185 275 360 550
30 62 32 54 33 35 PCBO
30 6.8 75 35 31 22 EMO1H
29 34 18 24 25 15 EMO1L
42 8.7 19 35 13 0 EMO7H
2.4 12 2.8 7.2 13 0.2 EMO7L
34 0 0.71 0.71 0 0 MRSM
Quantile
0–16.7%
16.7–33%
33–50%
50–66.7%
66.7–83%
83–100%
C
Study day
–1 90 185 275 360 550
9.3 11 16 18 10 9.5 PCBO
5.4 7.7 9.3 26 4.3 4 EMO1L
16 2.1 5 4.7 4.4 0 EMO7H
8.9 0 0.43 0.14 0 0 MRSM
0.29 7.6 1.6 0.8 3.6 0 EMO7L
9.3 2.3 17 3.5 8 6.8 EMO1H
D
Study day
–1 90 185 275 360 550
0 13 11 0.86 10 3.7 EMO7H
1.6 2.6 13 0 2.4 0.67 MRSM
0.14 11 2.9 1.4 5.6 14 PCBO
11 34 12 7.2 6.8 4.7 EMO1H
0.29 0.8 33 0 2 0 EMO7L
0.43 0.29 25 1.6 4.7 0.14 EMO1L

Figure H. Effects of emodepside on adult female worm motility.

(a) Mean worm motility values over time by treatment group.

(b) Worm motility values by animal and mean group values over time in the multiple high-dose emodepside group.

Worm motility score: 0 = none; 1 = intermediate; 2 = normal. No statistical comparisons were made.

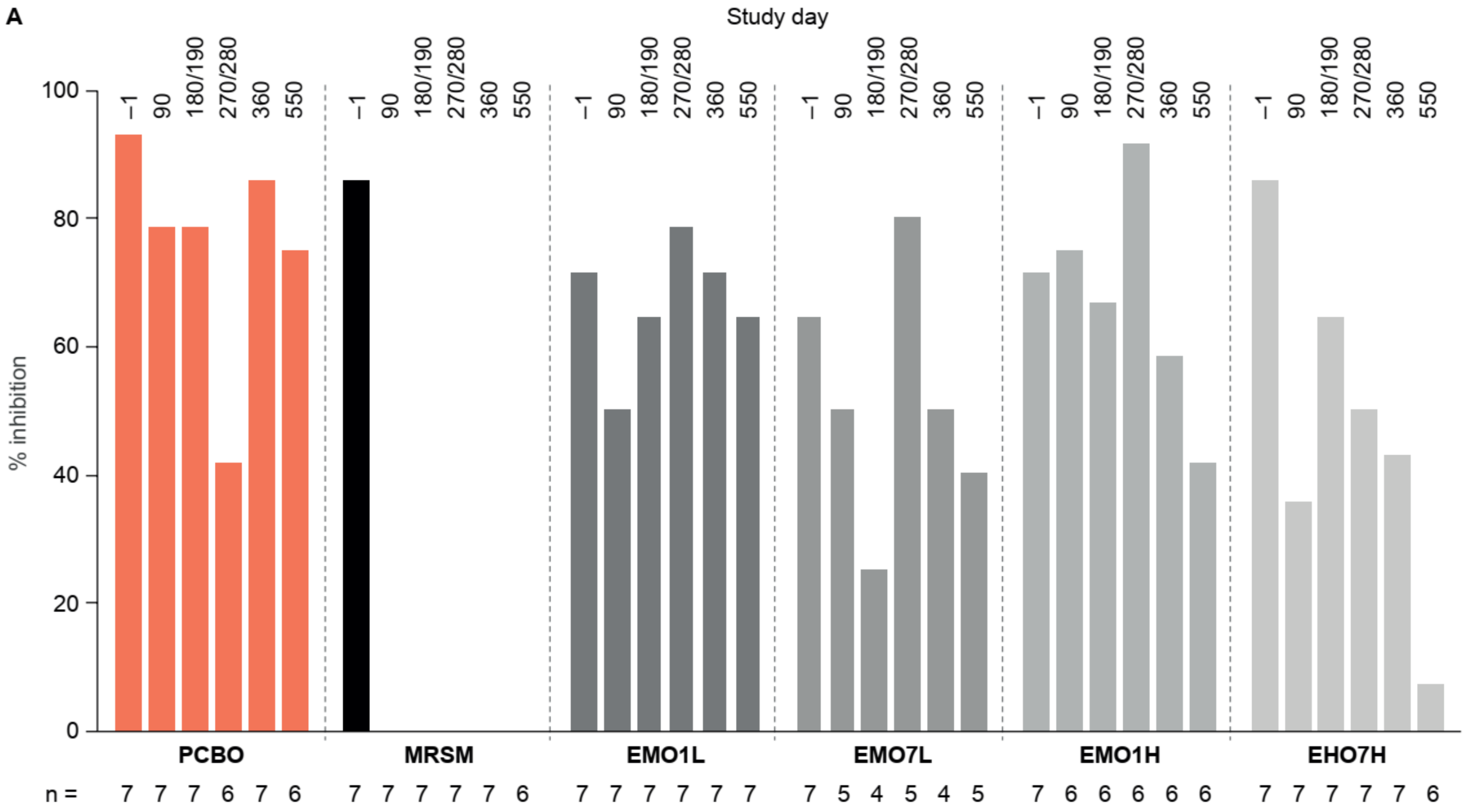

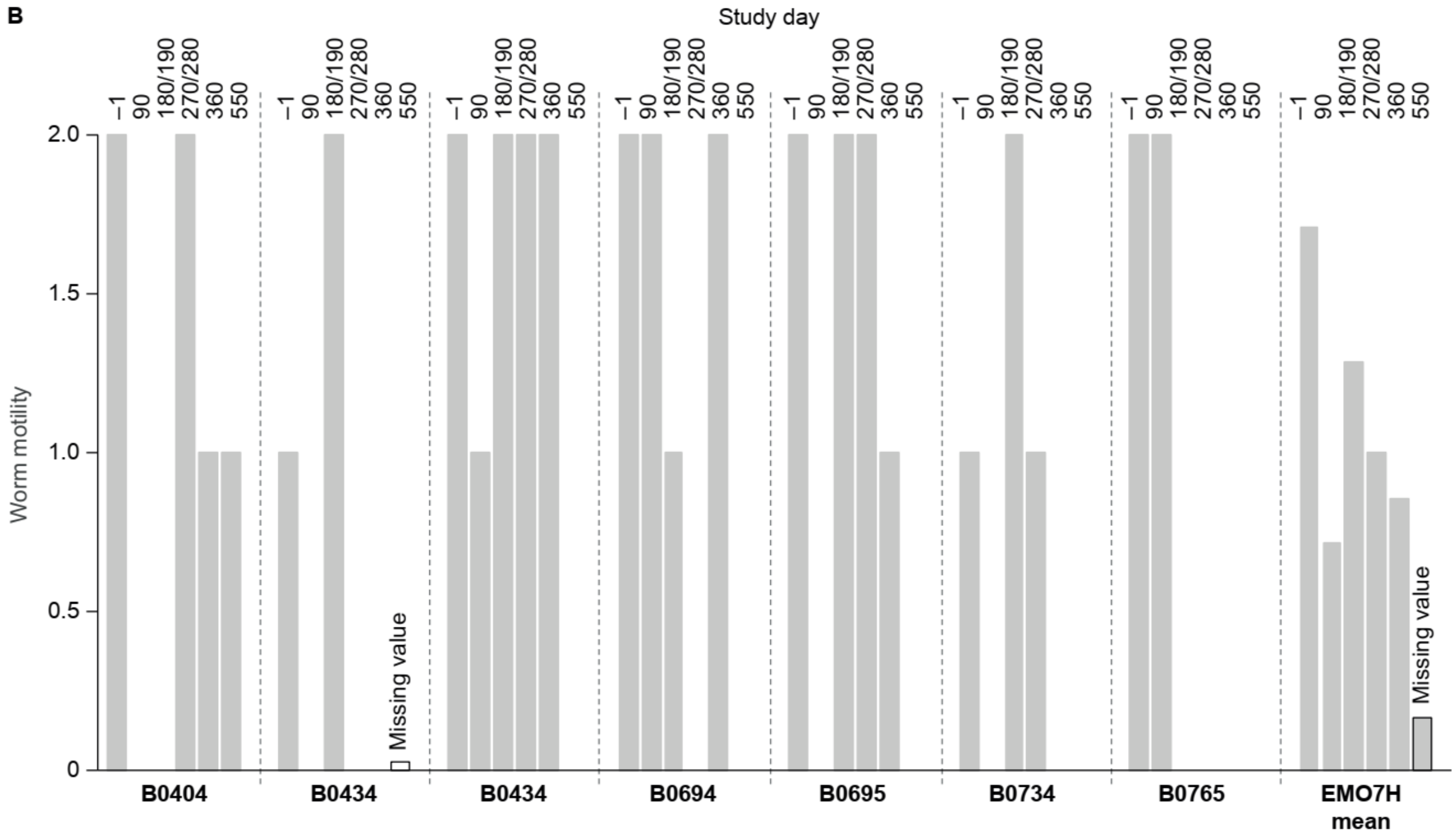

EMO1H, emodepside, 0.75 mg/kg, single dose; EMO7H, emodepside, 0.75 mg/kg, daily for seven days; EMO1L, emodepside, 0.15 mg/kg, single dose; EMO7L, emodepside, 0.15 mg/kg, daily for seven days; MRSM, melarsomine, 4 mg/kg, every other day for three days; PCBO, placebo.

Figure I. Effects of emodepside on adult female worm viability (MTT reduction).

(a) Summary data in comparison with melarsomine and placebo

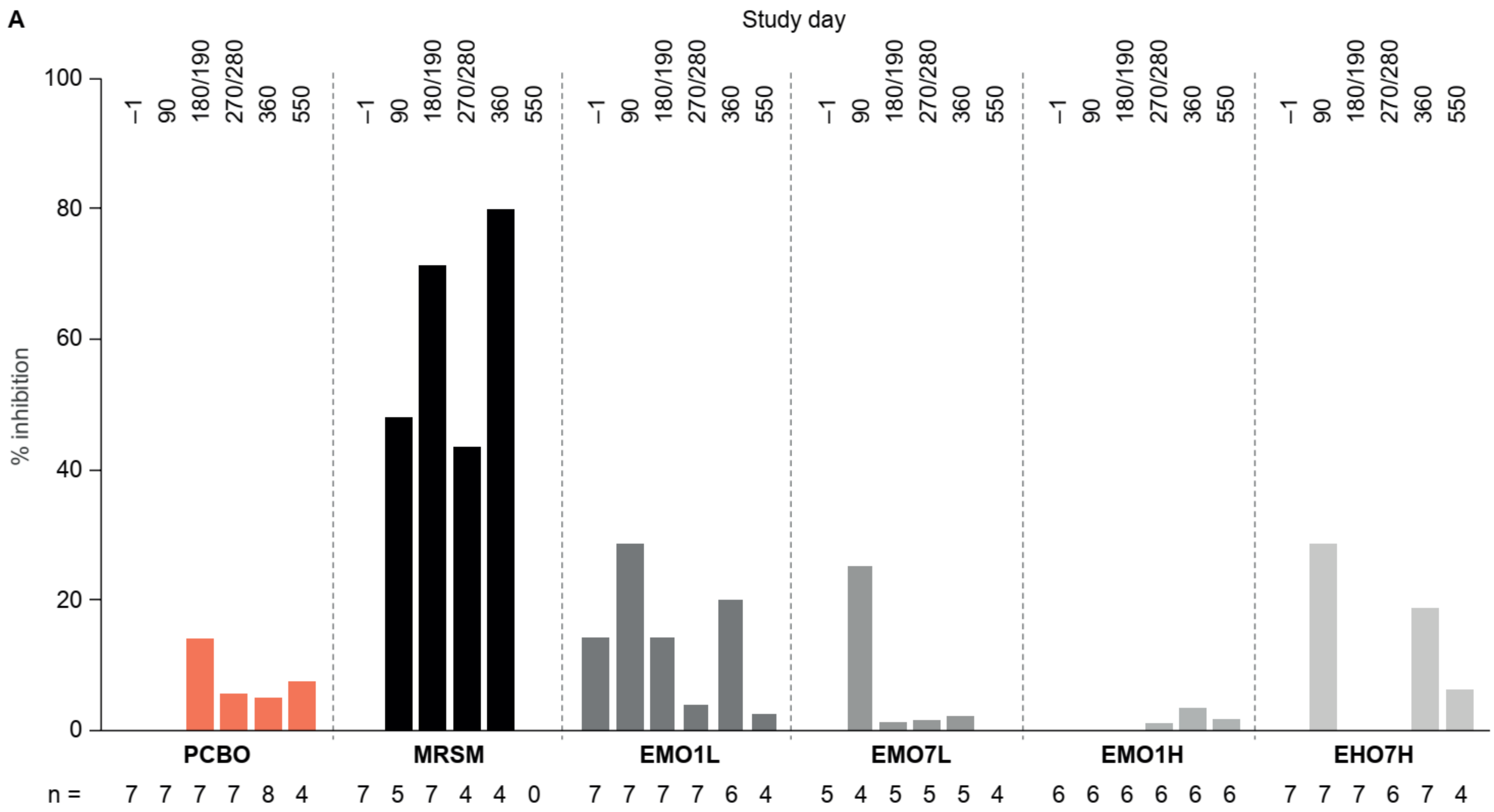

(b) Data for individual animals in the multiple high-dose emodepside group. No statistical comparisons were made.

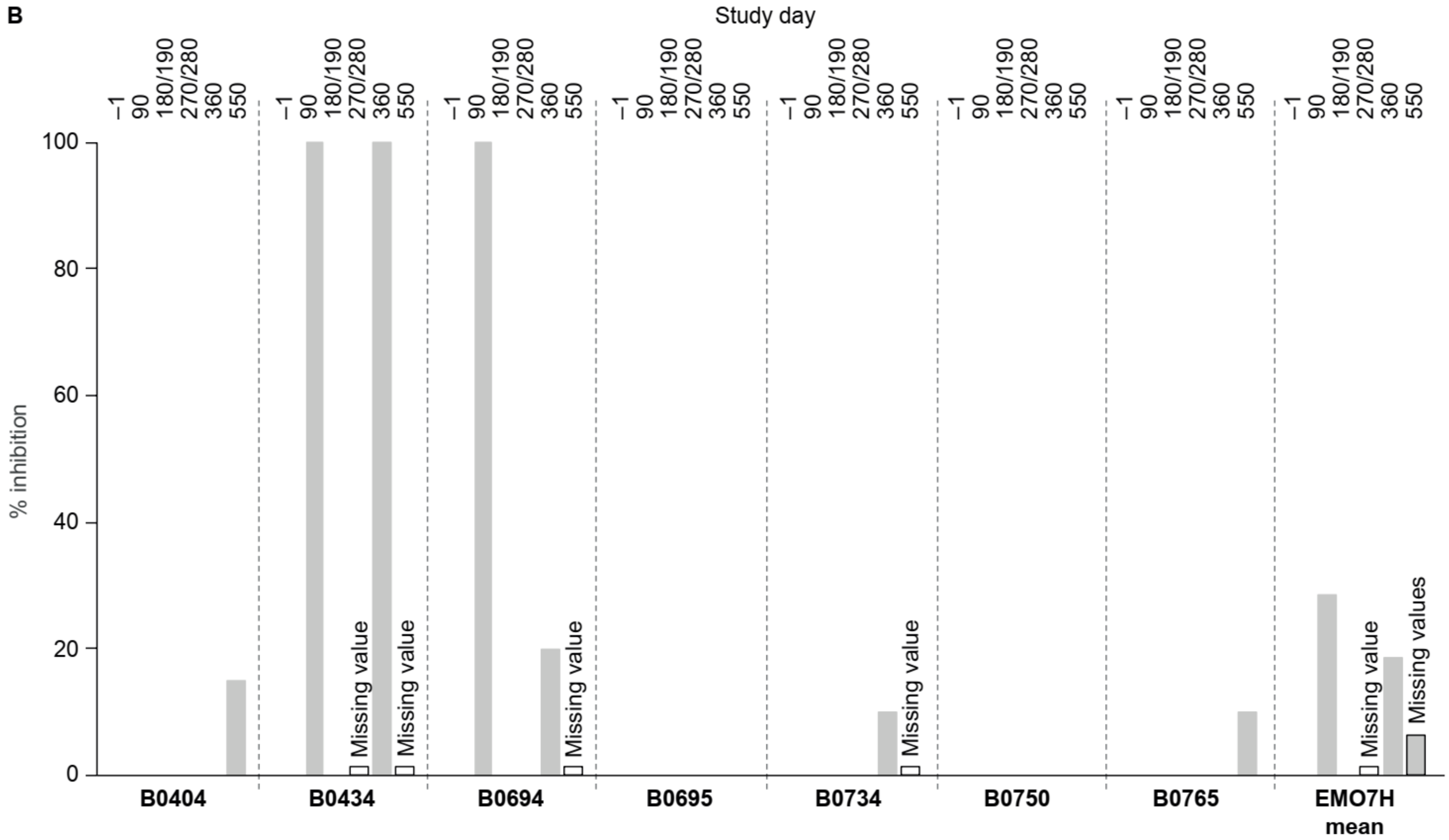

EMO1H, emodepside, 0.75 mg/kg, single dose; EMO7H, emodepside, 0.75 mg/kg, daily for seven days; EMO1L, emodepside, 0.15 mg/kg, single dose; EMO7L, emodepside, 0.15 mg/kg, daily for seven days; MRSM, melarsomine, 4 mg/kg, every other day for three days; PCBO, placebo.

Figure J. Effects of emodepside on mean adult female worm fecundity in comparison with melarsomine and placebo.

No statistical comparisons were made.

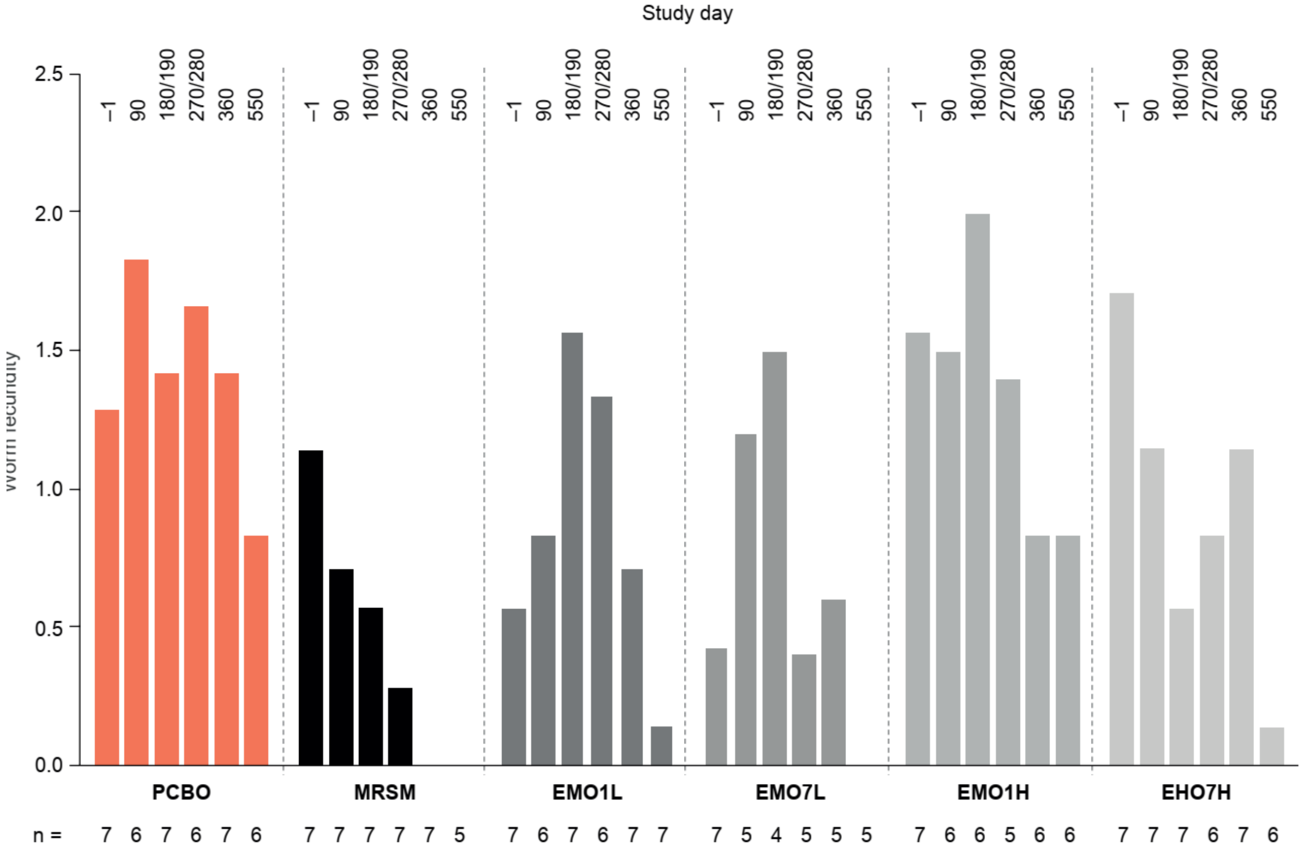

EMO1H, emodepside, 0.75 mg/kg, single dose; EMO7H, emodepside, 0.75 mg/kg, daily for seven days; EMO1L, emodepside, 0.15 mg/kg, single dose; EMO7L, emodepside, 0.15 mg/kg, daily for seven days; MRSM, melarsomine, 4 mg/kg, every other day for three days; PCBO, placebo.

Figure K. Effects of emodepside on mean adult male worm motility in comparison with melarsomine and placebo.

Worm motility score: 0 = none; 1 = intermediate; 2 = normal. No statistical comparisons were made.

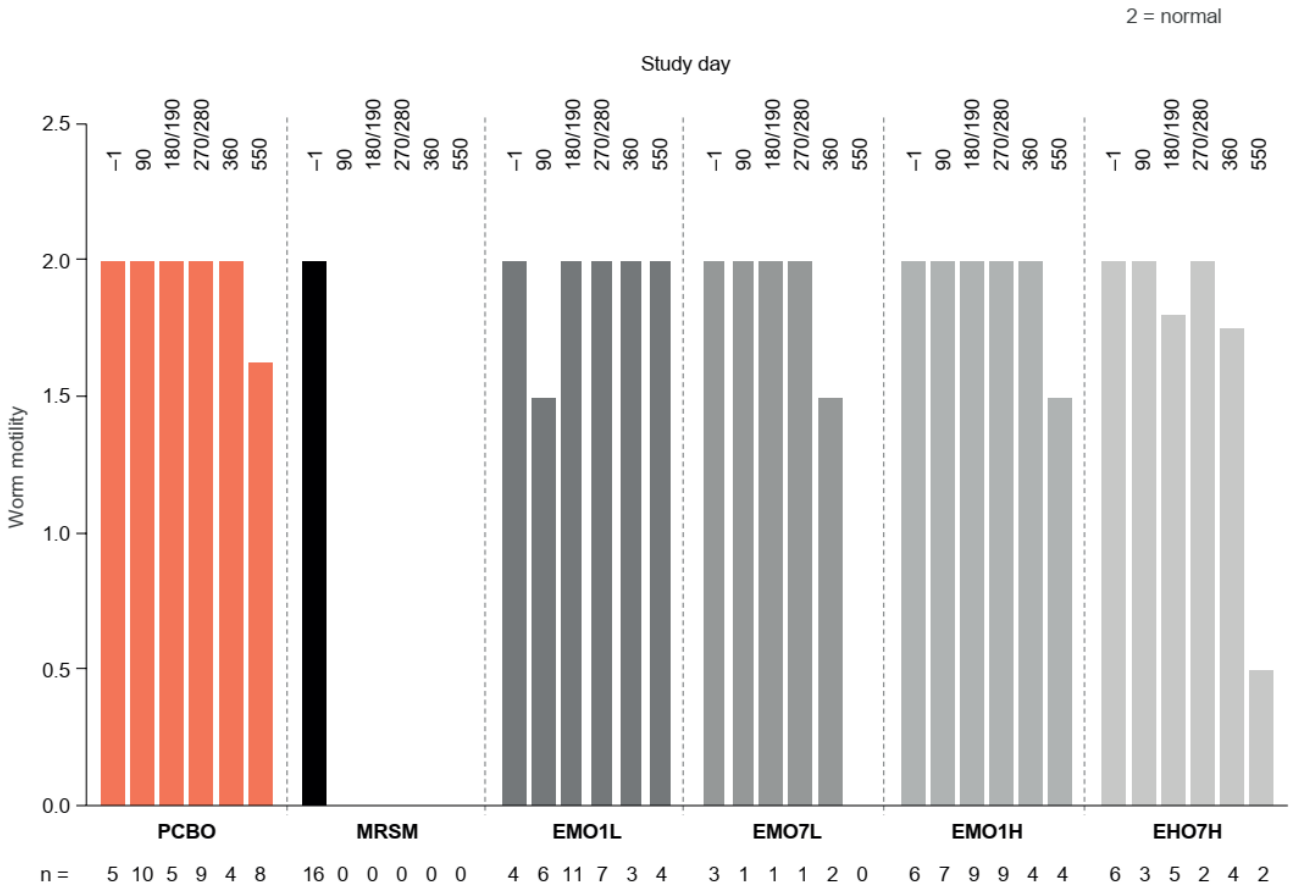

EMO1H, emodepside, 0.75 mg/kg, single dose; EMO7H, emodepside, 0.75 mg/kg, daily for seven days; EMO1L, emodepside, 0.15 mg/kg, single dose; EMO7L, emodepside, 0.15 mg/kg, daily for seven days; MRSM, melarsomine, 4 mg/kg, every other day for three days; PCBO, placebo.

Figure L. Histopathology of *Onchocerca ochengi* nodules treated with emodepside.

Micrographs (scale bars, 100 µm) show representative sections for experimental groups at day 290.

(a) EMO1L: Transverse and oblique section from a viable female (animal ID B0700, score 6; H&E stain).

(b) EMO7L: Transverse sections from a viable, productive female in a nodule with marked polymorpho-nuclear cell infiltrate and cuticle-associated Splendore–Hoeppli deposits (ID B0761, score 8; H&E stain).

(c) EMO1H: Transverse sections of viable, productive female with moderate polymorphonuclear cell infiltrate and cuticle-associated Splendore–Hoeppli deposits. Section of viable male parasite (M; score 5) also visible (ID B0439; score 5; H&E stain).

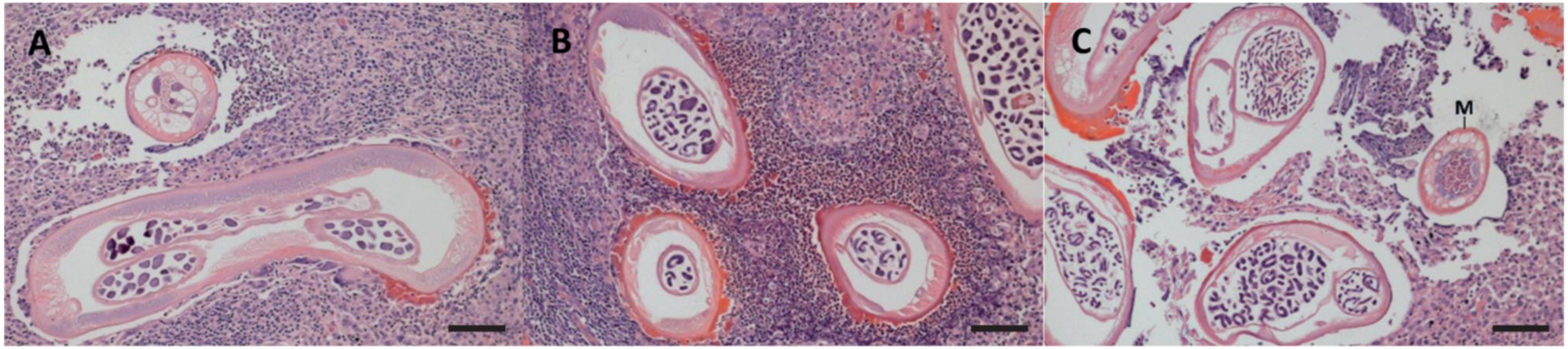

EMO1L, emodepside, 0.15 mg/kg, single dose; EMO7L, emodepside, 0.15 mg/kg, daily for seven days; EMO1H, emodepside, 0.75 mg/kg, single dose.

Figure M. Nodular polymorphonuclear counts per high-power field for each treatment group and timepoint.

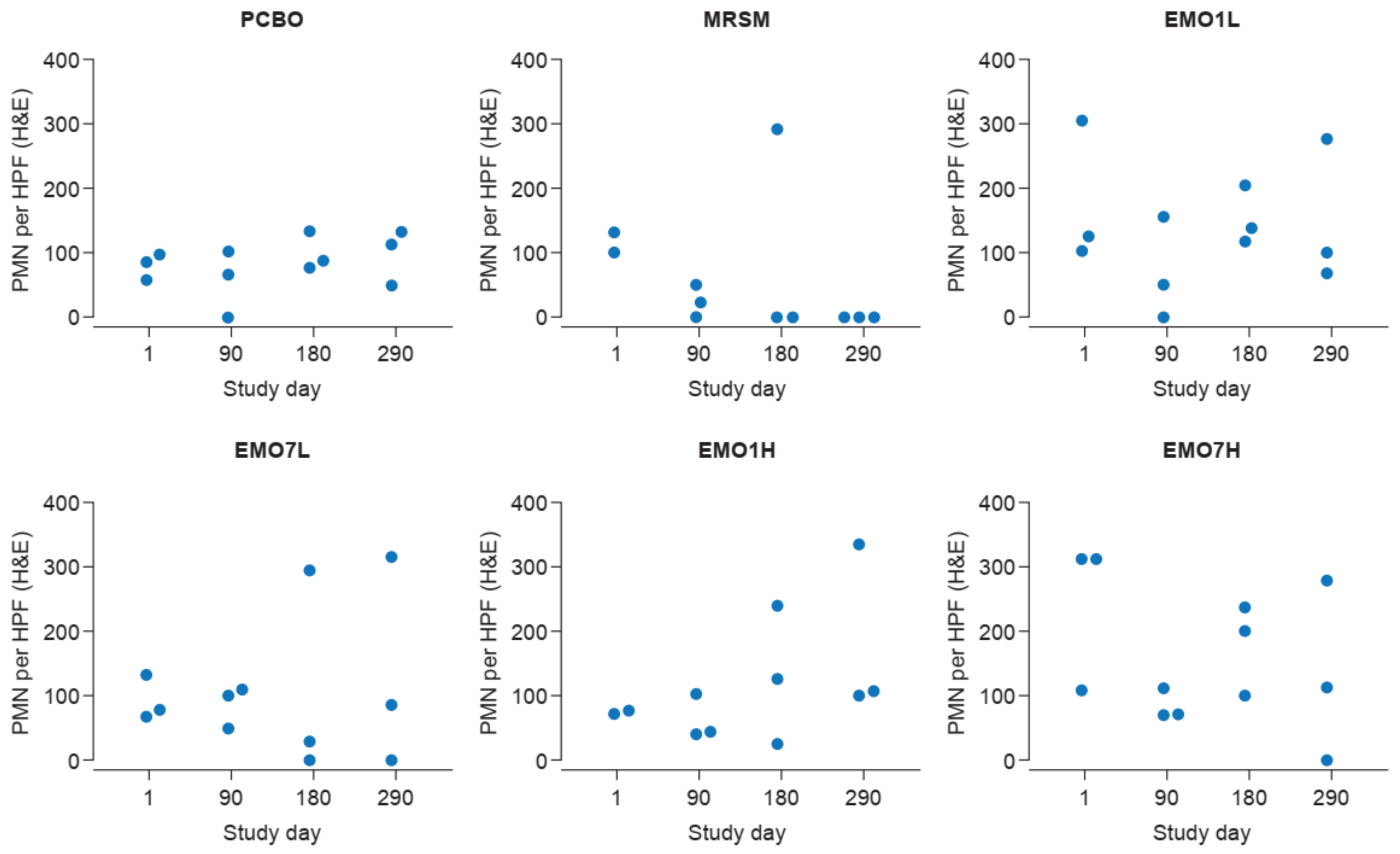

EMO1H, emodepside, 0.75 mg/kg, single dose; EMO7H, emodepside, 0.75 mg/kg, daily for seven days; EMO1L, emodepside, 0.15 mg/kg, single dose; EMO7L, emodepside, 0.15 mg/kg, daily for seven days; MRSM, melarsomine, 4 mg/kg, every other day for three days; PCBO, placebo.

Figure N. Nodular eosinophil counts as a percentage of total polymorphonuclear cell counts for each treatment group and timepoint.

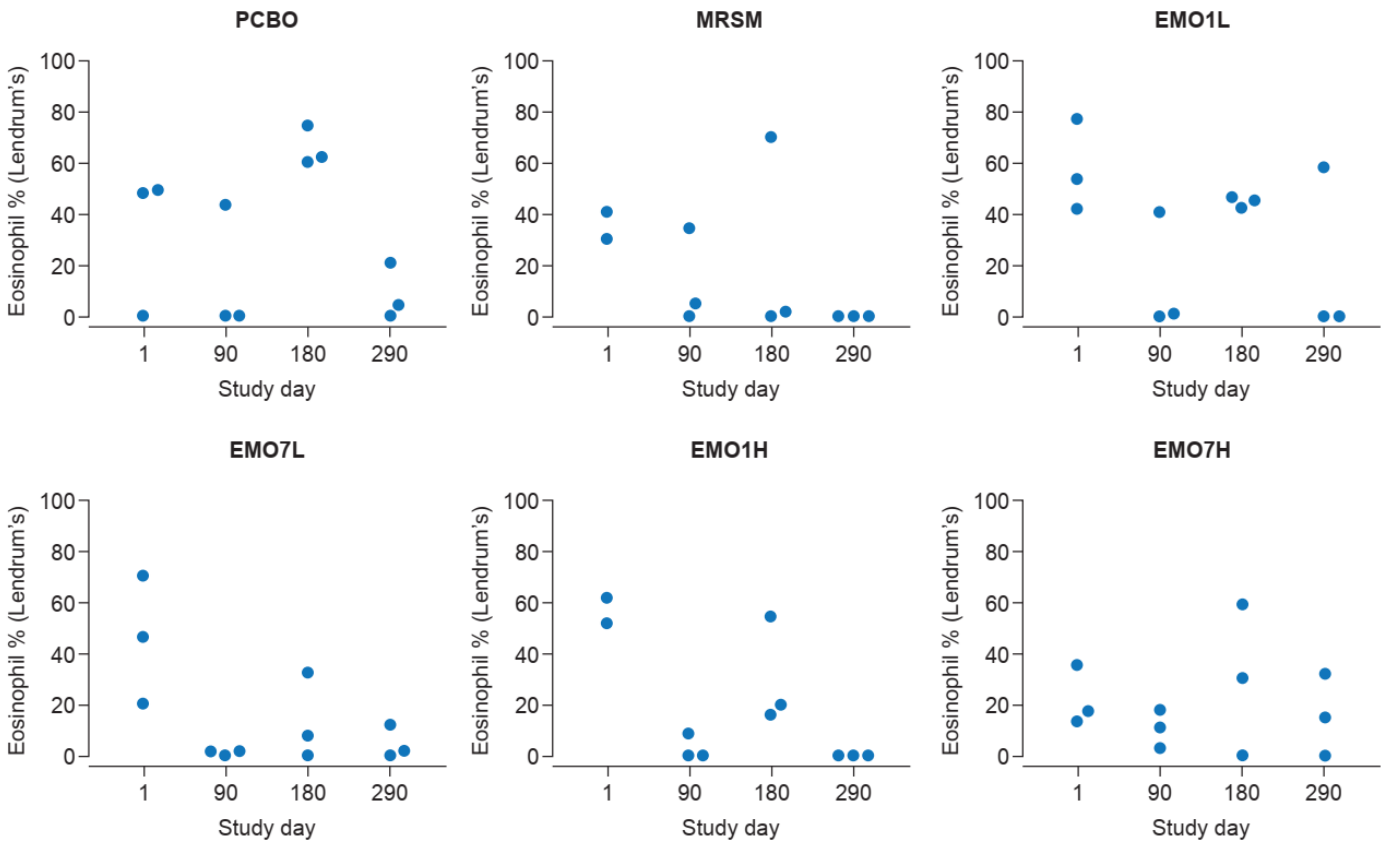

EMO1H, emodepside, 0.75 mg/kg, single dose; EMO7H, emodepside, 0.75 mg/kg, daily for seven days; EMO1L, emodepside, 0.15 mg/kg, single dose; EMO7L, emodepside, 0.15 mg/kg, daily for seven days; MRSM, melarsomine, 4 mg/kg, every other day for three days; PCBO, placebo.

Figure O. Linear model describing the relationship between change in adult male worm motility over time and total emodepside dose.

Intact male worms were removed from the worm mass and incubated alongside the anterior end of the female worms for 30 min at 37˚C for visual motility assessment on a three-point scale [1]. A linear model of worm motility over time for a given treatment group was estimated and repeated for each group; the slope β is reported in this figure. The null hypothesis was β = 0, and *t* statistics were used to reject the null hypothesis and to estimate the *P* value. Datapoints are mean values and error bars represent SEM. The dashed line in red indicates the mean for the positive control (MRSM). The SEM for the positive control was very small (± 3.3×10^{-6}) and is essentially superimposed on the mean. A regression model found a significant relationship between dose and decrease in motility (black line) (*P* = 0.003). EMO1H, emodepside, 0.75 mg/kg, single dose; EMO7H, emodepside, 0.75 mg/kg, daily for seven days; EMO1L, emodepside, 0.15 mg/kg, single dose; EMO7L, emodepside, 0.15 mg/kg, daily for seven days; MRSM, melarsomine, 4 mg/kg, every other day for three days; PCBO, placebo.

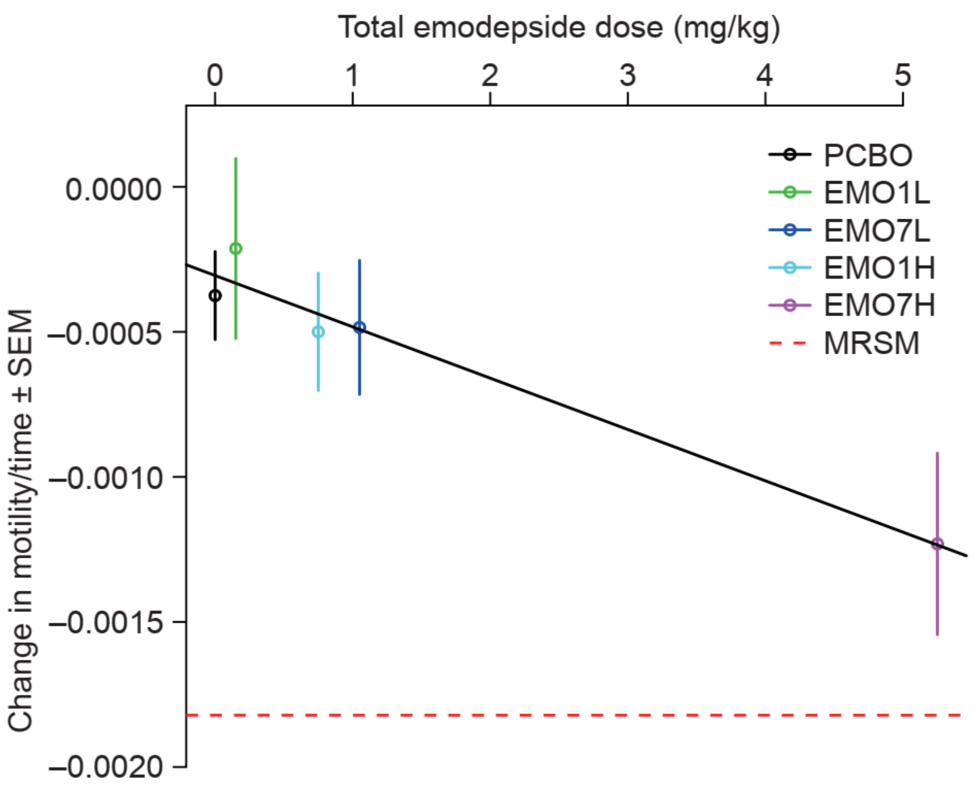

Table A. Grading and scoring system for histopathological assessment of *Onchocerca ochengi* nodules.

**Grading of specific anatomical features**	**Pathology score**
A: Not observed in any parasite sections examined	1: No evidence of damaged/degraded tissue
B: Observed in section, but not abundant (<50% of parasite sections examined)	2: Evidence of damage/degradation tissue
C: Observed frequently (>50% of parasite sections examined)	3: Extensive tissue damage/degradation

Table B. Summary of PK parameters of non-compartmental analysis of the simulated concentration-time profiles after repeated administration of emodepside.

**Dose**	**mg/kg**	**0.15**	**0.75**
AUC (0–24)	h.µg/L	515	5,008
AUC (120–144)	h.µg/L	857	7,290
C_{max}	µg/L	122	2,369
$C_{max,ss}$	µg/L	138	2,425

AUC, area under the curve for selected time interval; C_{max} maximum concentration after first dose; $C_{max,ss}$ maximum concentration at steady state.

Table C. Observed emodepside C_{max} and T_{max} in cattle.

Animal	Group	Observed in plasma						Model				Observed in skin			
		Time	C_{max} (µg/kg)	t_{last}	Conc. at t_{last} (µg/kg)	Lowest C_{max} (µg/kg)	Highest C_{max} (µg/kg)	C_{max} (µg/kg)	t_{max}	AUC (µg/kg*h)	$t_{1/2}$ (h)	t_{max}	C_{max} (µg/kg)	t_{last}	Conc. at t_{last} (µg/kg)
B0696	EMO1L	5 min	456	168 h	2.17						71.1	24 h	136.2	24 h	136.2
B0698	EMO1L	30 min	226	168 h	1.68						90.2	24 h	94.8	24 h	94.8
B0700	EMO1L	5 min	492	336 h	1.33						95.7	24 h	770	24 h	770
B0722	EMO1L	5 min	562	504 h	1.05	226	1150	352	5 min	3259	163.3	–	–	–	–
B0730	EMO1L	5 min	527	168 h	2.38						52.3	24 h	149	24 h	149
B0731	EMO1L	5 min	270	168 h	1.29						78.7	24 h	197.4	168 h	120.6
B0762	EMO1L	5 min	1150	336 h	1.13						100.8	24 h	89.6	24 h	89.6
B0228	EMO7L	24 h	999	336 h	5.61						44.8	168 h	183.4	168 h	183.4
B0407	EMO7L	24 h	947	504 h	20.8						139.1	24 h	88.6	336 h	29.4
B0549	EMO7L	5 min	547	504 h	3.97						116.9	24 h	160	24 h	160
B0725	EMO7L	5 min	472	504 h	3	435	999	401	144 h	24041	100.6	24 h	224	168 h	38.8
B0733	EMO7L	5 min	723	504 h	4.62						151.9	24 h	39.8	504 h	28.2
B0735	EMO7L	48 h	435	504 h	3.77						81.7	168 h	91.6	336 h	34.8
B0761	EMO7L	24 h	683	504 h	4.35						117.1	168 h	50.2	336 h	28.2
B0054	EMO1H	5 min	2021	168 h	5.21						51.7	24 h	111.4	24 h	111.4
B0405	EMO1H	5 min	2714	504 h	5.2						263.1	24 h	61	24 h	61
B0439	EMO1H	5 min	1459	504 h	3.37						157.4	24 h	171.4	168 h	21
B0721	EMO1H	24 h	1476	504 h	1.94	1459	2714	1759	5 min	16295	99.8	168 h	161	336 h	27
B0726	EMO1H	5 min	2383	504 h	2.11						98.4	24 h	112.2	168 h	39.6
B0727	EMO1H	24 h	2325	168 h	30.7						65.3	24 h	124.8	504 h	20.4
B0728	EMO1H	5 min	1896	336 h	1.55						70.5	24 h	828	24 h	828
B0404	EMO7H	24 h	1715	504 h	34.1						139.5	24 h	426	672 h	24.4
B0434	EMO7H	5 min	2085	504 h	17.2						107.2	24 h	113.6	336 h	30
B0694	EMO7H	72 h	2701	504 h	41.1						122.3	24 h	548	336 h	39.2
B0695	EMO7H	5 min	2900	504 h	29.4	1417	2900	2003	144 h	120205	76.7	24 h	958	336 h	58.2
B0734	EMO7H	5 min	1417	504 h	4.03						56.4	24 h	1062	168 h	106
B0750	EMO7H	72 h	2346	504 h	22.4						103.0	24 h	452	336 h	38
B0765	EMO7H	168 h	2325	504 h	29						53.1	24 h	486	2160 h	55.2

Table D. Summary statistics for histological sections examined by experimental group and timepoint.

Sections were prepared from three nodules per experimental group per timepoint (n = 72)

**Day**		**1**	**90**	**180**	**290**
**PCBO**	Females	3	3	3	3
	Males	2	0	2	0
	Degenerate nodule*	0	0	0	0
**MRSM**	Females	2	2	1	0
	Males	2	0	0	0
	Degenerate nodule	1	1	2	3
**EMO1L**	Females	3	2	3	3
	Males	1	1	0	1
	Degenerate nodule	0	1	0	0
**EMO7L**	Females	3	3	2	2
	Males	1	1	0	0
	Degenerate nodule	0	0	1	1
**EMO1H**	Females	2	3	3	3
	Males	1	0	1	2
	Degenerate nodule	0	0	0	0
**EMO7H**	Females	3	3	3	2
	Males	0	0	0	0
	Degenerate nodule	0	0	0	1

*Degenerate nodules are as those containing either no parasite sections or only cuticular fragments.

EMO1H, emodepside, 0.75 mg/kg, single dose; EMO7H, emodepside, 0.75 mg/kg, daily for seven days; EMO1L, emodepside, 0.15 mg/kg, single dose; EMO7L, emodepside, 0.15 mg/kg, daily for seven days; MRSM, melarsomine, 4 mg/kg, every other day for three days; PCBO, placebo.

Table E. Mean histopathological scores (graded 1–4) for each specific anatomical component by treatment group and timepoint.

		Study day			
**Anatomical feature**		**1**	**90**	**180**	**290**
**Cuticle**	**PCBO**	1.00	2.00	1.40	1.67
	**MRSM**	1.60	3.00	3.33	4.00
	**EMO1L**	1.50	1.83	1.67	2.25
	**EMO7L**	1.00	1.25	2.67	2.33
	**EMO1H**	1.75	1.00	1.50	1.20
	**EMO7H**	1.33	1.33	1.67	2.00
**Hypodermis**	**PCBO**	1.00	1.33	1.20	1.67
	**MRSM**	1.60	3.33	3.00	4.00
	**EMO1L**	1.00	2.17	1.00	1.50
	**EMO7L**	1.00	1.00	2.33	2.33
	**EMO1H**	2.00	1.00	1.00	1.00
	**EMO7H**	1.00	1.33	1.67	2.33
**Longitudinal muscle**	**PCBO**	1.00	1.00	1.00	1.00
	**MRSM**	1.60	2.67	3.00	4.00
	**EMO1L**	1.00	1.50	1.00	1.00
	**EMO7L**	1.00	1.00	2.00	2.00
	**EMO1H**	1.75	1.33	1.25	1.20
	**EMO7H**	1.00	1.33	1.33	2.33
**Pseudocoleomic cavity**	**PCBO**	1.00	1.33	1.20	2.00
	**MRSM**	1.60	3.33	3.33	4.00
	**EMO1L**	1.00	2.00	1.33	1.50
	**EMO7L**	1.50	1.75	2.67	2.33
	**EMO1H**	1.75	1.33	1.50	1.20
	**EMO7H**	1.33	1.33	2.00	2.00
**Gut**	**PCBO**	1.00	1.00	1.00	1.00
	**MRSM**	1.60	3.33	3.00	4.00
	**EMO1L**	1.00	1.83	1.00	1.25
	**EMO7L**	1.00	1.00	2.00	2.00
	**EMO1H**	1.75	1.00	1.00	1.00
	**EMO7H**	1.00	1.00	1.00	2.00

EMO1H, emodepside, 0.75 mg/kg, single dose; EMO7H, emodepside, 0.75 mg/kg, daily for seven days; EMO1L, emodepside, 0.15 mg/kg, single dose; EMO7L, emodepside, 0.15 mg/kg, daily for seven days; MRSM, melarsomine, 4 mg/kg, every other day for three days; PCBO, placebo.

Table F. Linear mixed-effects model results for cumulative histopathological worm scores analysed (a) at each timepoint compared with the placebo control group and (b) within each treatment group compared with pre-treatment observations (day 1).

Coefficient values (and standard error) values indicate expected degree of increase or decrease relative to respective reference groups. P-values are corrected for multiple testing (*, significant; **highly significant).

(a)

Group	Study day							
	**1**		**90**		**180**		**290**	
	Coefficient (SE)	P-value	Coefficient (SE)	P-value	Coefficient (SE)	P-value	Coefficient (SE)	P-value
**MRSM**	<0.01 (0.45)	1.00	8.00 (2.01)	<0.004**	7.31 (2.41)	<0.03*	8.67 (2.97)	<0.03*
**EMO1L**	0.50 (0.45)	0.58	2.75 (1.94)	0.36	0.31 (2.41)	1.00	0.63 (2.95)	1.00
**EMO7L**	0.59 (0.45)	0.42	–3.33 (1.98)	1.00	4.64 (2.41)	0.16	2.33 (2.97)	0.89
**EMO1H**	0.59 (0.49)	1.00	–6.67 (2.01)	1.00	0.77 (2.38)	1.00	–1.82 (2.94)	1.00
**EMO7H**	0.67 (0.46)	0.36	1.58 (2.01)	1.00	1.97 (2.41)	1.00	2.00 (2.97)	1.00

(b)

Study day	Group											
	**PCBO**		**MRSM**		**EMO1L**		**EMO7L**		**EMO1H**		**EMO7H**	
	Coefficient (SE)	P-value	Coefficient (SE)	P-value	Coefficient (SE)	P-value	Coefficient (SE)	P-value	Coefficient (SE)	P-value	Coefficient (SE)	P-value
**90**	1.35 (0.57)	0.08	9.45 (1.81)	<0.003**	3.89 (1.56)	0.06	0.52 (2.00)	1	−0.002 (0.77)	1.00	0.67 (2.66)	1.00
**180**	0.80 (0.50)	0.14	8.12 (1.81)	<0.006**	1.04 (1.82)	1.00	4.58 (2.13)	0.13	0.68 (0.72)	0.75	2.00 (2.66)	0.96
**290**	2.35 (0.57)	<0.005**	11.11 (1.81)	<0.001**	2.94 (1.71)	0.25	3.91 (2.13)	0.21	−0.14 (0.70)	1.00	3.67 (2.67)	0.43

EMO1H, emodepside, 0.75 mg/kg, single dose; EMO7H, emodepside, 0.75 mg/kg, daily for seven days; EMO1L, emodepside, 0.15 mg/kg, single dose; EMO7L, emodepside, 0.15 mg/kg, daily for seven days; MRSM, melarsomine, 4 mg/kg, every other day for three days; PCBO, placebo; SE, standard error.

Table G. Linear mixed-effects model results for nodular polymorphonuclear counts per high-power field analysed (a) at each timepoint compared with the placebo control group and (b) within each treatment group compared with pre-treatment observations (day 1).

Coefficient values (and standard error) values indicate expected degree of increase or decrease relative to respective reference groups. P-values are corrected for multiple testing (*significant; **highly significant).

(a)

Group	Study day 1		Study day 90		Study day 180		Study day 290	
	Coefficient (SE)	P-value	Coefficient (SE)	P-value	Coefficient (SE)	P-value	Coefficient (SE)	P-value
**MRSM**	35.56 (68.04)	0.61	−31.96 (37.23)	0.41	−2.50 (90.62)	0.98	−98.33 (93.14)	0.31
**EMO1L**	97.31 (60.86)	0.14	12. 36 (37.23)	0.75	54.33 (90.62)	0.56	49.94 (93.14)	0.60
**EMO7L**	12.25 (60.86)	0.84	30.11 (37.23)	0.43	8.17 (90.62)	0.93	35.22 (93.14)	0.71
**EMO1H**	−5.69 (68.04)	0.94	6.04 (37.23)	0.87	31.17 (90.62)	0.74	82.17 (93.14)	0.40
**EMO7H**	163.97 (60.86)	< 0.02*	27.89 (37.23)	0.47	79.50 (90.62)	0.40	32.00 (93.14)	0.74

(b)

Study day	Group PCBO		Group MRSM		Group EMO1L		Group EMO7L		Group EMO1H		Group EMO7H	
	Coefficient (SE)	P-value	Coefficient (SE)	P-value	Coefficient (SE)	P-value	Coefficient (SE)	P-value	Coefficient (SE)	P-value	Coefficient (SE)	P-value
**90**	−23.81 (31.17)	0.47	−89.76 (81.56)	0.32	−108.75 (58.39)	0.11	−5.94 (66.90)	0.93	15.48 (57.69)	0.80	−159.89 (75.66)	0.08
**180**	19.31 (31.17)	0.56	−17.19 (81.56)	0.84	−23.67 (58.39)	0.70	15.22 (66.90)	0.83	83.71 (57.69)	0.21	−65.17 (75.66)	0.42
**290**	18.14 (31.17)	0.58	−114.19 (81.56)	0.22	−29.22 (58.39)	0.63	41.11 (66.90)	0.56	133.54 (57.69)	0.07	−133.83 (75.66)	0.18

EMO1H, emodepside, 0.75 mg/kg, single dose; EMO7H, emodepside, 0.75 mg/kg, daily for seven days; EMO1L, emodepside, 0.15 mg/kg, single dose; EMO7L, emodepside, 0.15 mg/kg, daily for seven days; MRSM, melarsomine, 4 mg/kg, every other day for three days; PCBO, placebo; SE, standard error.

Table H. Linear mixed-effects model results for nodular eosinophil counts as a percentage of total polymorphonuclear cell counts analysed (a) at each timepoint compared with the placebo control group and (b) within each treatment group compared with pre-treatment observations (day 1).

Coefficient and standard error values indicate the expected degree of increase or decrease relative to respective reference groups. P-values are corrected for multiple testing (*, significant; **highly significant).

(a)

Group	Study day							
	1		90		180		290	
	Coefficient (SE)	P-value	Coefficient (SE)	P-value	Coefficient (SE)	P-value	Coefficient (SE)	P-value
**MRSM**	3.09 (17.98)	1.00	–1.40 (13.34)	1.00	–41.88 (19.04)	0.10	–8.47 (13.17)	1.00
**EMO1L**	25.15 (16.09)	0.30	–0.72 (13.34)	1.00	–20.99 (19.04)	0.58	11.01 (13.17)	0.82
**EMO7L**	12.92 (16.09)	0.88	–13.39 (13.34)	0.67	–52.41 (19.04)	<0.04*	–3.96 (13.17)	1.00
**EMO1H**	24.35 (17.98)	0.41	–11.68 (13.34)	0.80	–35.80 (19.04)	0.17	–8.47 (13.17)	1.00
**EMO7H**	–10.43 (16.09)	1.00	–4.02 (13.34)	1.00	–35.97 (19.04)	0.17	7.25 (13.17)	1.00

(b)

Study day	Group											
	PCBO		MRSM		EMO1L		EMO7L		EMO1H		EMO7H	
	Coefficient (SE)	P-value	Coefficient (SE)	P-value	Coefficient (SE)	P-value	Coefficient (SE)	P-value	Coefficient (SE)	P-value	Coefficient (SE)	P-value
**90**	–18.01 (16.40)	0.63	–19.20 (17.65)	0.65	–43.89 (18.23)	0.11	–44.34 (12.68)	<0.03*	–54.05 (10.86)	<0.01*	–11.62 (13.89)	0.87
**180**	33.22 (16.40)	0.18	–8.42 (17.65)	1.00	–12.91 (18.23)	1.00	–32.11 (12.68)	0.09	–26.92 (10.86)	0.11	7.68 (13.89)	1.00
**290**	–24.11 (16.40)	0.38	–32.35 (17.65)	0.25	–38.25 (18.23)	0.16	–40.99 (12.68)	<0.04*	–56.93 (10.86)	<0.01*	–6.43 (13.89)	1.00

EMO1H, emodepside, 0.75 mg/kg, single dose; EMO7H, emodepside, 0.75 mg/kg, daily for seven days; EMO1L, emodepside, 0.15 mg/kg, single dose; EMO7L, emodepside, 0.15 mg/kg, daily for seven days; MRSM, melarsomine, 4 mg/kg, every other day for three days; PCBO, placebo; SE, standard error

Table I. Fraction of normal adult worm motility (% [*n*]) by treatment group, sex, and timepoint.

Group	Study day											
	–1		90		185		275		360		550	
	♀	♂	♀	♂	♀	♂	♀	♂	♀	♂	♀	♂
**PCBO**	86 (7)	100 (5)	71 (7)	100 (10)	71 (7)	100 (5)	40 (5)	100 (9)	71 (7)	100 (4)	50 (6)	63 (8)
**MRSM**	71 (7)	100 (16)	0 (7)	–	0 (7)	–	0 (7)	–	0 (2)	–	0 (2)	0 (1)
**EMO1L**	57 (7)	100 (4)	29 (7)	50 (6)	43 (7)	100 (11)	71 (7)	100 (7)	43 (7)	80 (5)	29 (7)	100 (5)
**EMO7L**	43 (7)	100 (3)	40 (5)	100 (1)	0 (4)	100 (1)	67 (6)	100 (1)	25 (4)	67 (3)	0 (5)	–
**EMO1H**	57 (7)	100 (6)	50 (6)	100 (7)	33 (6)	100 (9)	83 (6)	100 (8)	20 (5)	100 (4)	17 (6)	50 (4)
**EMO7H**	71 (7)	100 (6)	29 (7)	100 (3)	57 (7)	80 (5)	43 (7)	100 (2)	29 (7)	75 (4)	0 (6)	0 (2)

Empty cells indicate occasions when no male worms were present. EMO1H, emodepside, 0.75 mg/kg, single dose; EMO7H, emodepside, 0.75 mg/kg, daily for seven days; EMO1L, emodepside, 0.15 mg/kg, single dose; EMO7L, emodepside, 0.15 mg/kg, daily for seven days; MRSM, melarsomine, 4 mg/kg, every other day for three days; PCBO, placebo.

Reference

1. Renz A, Trees AJ, Achu-Kwi D, Edwards G, Wahl G. Evaluation of suramin, ivermectin and CGP 20376 in a new macrofilaricidal drug screen, *Onchocerca ochengi* in African cattle. Trop Med Parasitol. 1995;46(1):31-7. Epub 1995/03/01. PubMed PMID: 7631125.
